# The vertebrate small leucine-rich proteoglycans: amplification of a clustered gene family and evolution of their transcriptional profile in jawed vertebrates

**DOI:** 10.1093/g3journal/jkaf003

**Published:** 2025-01-08

**Authors:** Nathan Gil, Nicolas Leurs, Camille Martinand-Mari, Mélanie Debiais-Thibaud

**Affiliations:** Institut des Sciences de l’Evolution de Montpellier, Université de Montpellier, CNRS, IRD, 34090 Montpellier, France; Institut des Sciences de l’Evolution de Montpellier, Université de Montpellier, CNRS, IRD, 34090 Montpellier, France; Institut des Sciences de l’Evolution de Montpellier, Université de Montpellier, CNRS, IRD, 34090 Montpellier, France; Institut des Sciences de l’Evolution de Montpellier, Université de Montpellier, CNRS, IRD, 34090 Montpellier, France

**Keywords:** genomic evolution, evolution of gene expression, gnathostomes, cartilaginous fishes, small-spotted catshark, SLRP, skeleton evolution

## Abstract

Small Leucine-Rich Proteoglycans (SLRPs) are a major family of vertebrate proteoglycans. In bony vertebrates, SLRPs have a variety of functions from structural to signaling and are found in extracellular matrices, notably in skeletal tissues. However, there is little or no data on the diversity, function and expression patterns of SLRPs in cartilaginous fishes, which hinders our understanding of how these genes evolved with the diversification of vertebrates, in particular regarding the early events of whole-genome duplications that shaped gnathostome and cyclostome genomes. We used a selection of chromosome-level assemblies of cartilaginous fish and other vertebrate genomes for phylogeny and synteny reconstructions, allowing better resolution and understanding of the evolution of this gene family in vertebrates. Novel SLRP members were uncovered together with specific loss events in different lineages. Our reconstructions support that the canonical SLRPs have originated from different series of tandem duplications that preceded the extant vertebrate last common ancestor, one of them even preceding the extant chordate last common ancestor. They then further expanded with additional tandem and whole-genome duplications during the diversification of extant vertebrates. Finally, we characterized the expression of several SLRP members in the small-spotted catshark *Scyliorhinus canicula* and from this, inferred conserved and derived SLRP expression in several skeletal and connective tissues in jawed vertebrates.

## Graphical Abstract

**Graphical Abstract:**
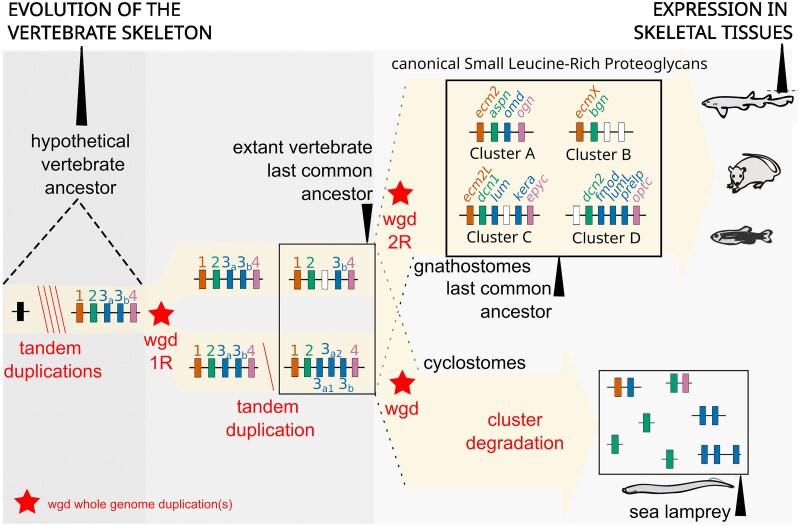

## Teaser

The evolution of vertebrates occurred more than 500 Mya with the development of a cartilaginous skeleton composed of a diversity of proteins that include proteoglycans such as the Small Leucine-Rich Proteoglycans (SLRPs). Gil *et al*. identified how the SLRP family expanded from one to more than twenty genes in the course of vertebrate evolution, over several events of gene tandem duplication and whole genome duplication events, generating canonical SLRP gene cluster. The authors’ gene expression data support their ancestral function in vertebrate skeletal development.

## Introduction

A diversity of connective tissues has emerged with the evolution of vertebrates, including their skeletal tissues (Root *et al*. 2021, 2022). The macromolecular content of their extracellular matrices (ECM) consists firstly of collagen fibers (e.g. Collagen type I in bone and Collagen type II in cartilage), together with other types of proteins, and lipids. Among non-collagenous proteins, proteoglycans are particularly abundant in highly hydrated ECMs (proteoglycan content in cartilage: 5–7% w/w; Hardingham 2006). Within proteoglycans, the largest known vertebrate family is the Small Leucine-Rich Proteoglycans (SLRPs). In this family, the protein moiety is relatively small (36–42 kDa) and has a distinctive leucine-rich repeat (LRR) domain (Nikitovic *et al*. 2012; Iozzo and Schaefer 2015). They can be bound to any of the 3 glycosaminoglycans (Zappia *et al*. 2020): heparin/heparan sulfate (HS), chondroitin/dermatan sulfate (C/DS) and keratan sulfate (KS). SLRPs play critical roles in the structure and assembly of various ECMs and hence in the development, structure, and homeostasis of connective tissues (Hardingham 2006; Park *et al*. 2008; Boskey 2010; Nikitovic *et al*. 2012). For instance, they are known to interact with several types of collagen fibers by regulating their assembly via their protein moiety, while their glycosaminoglycans control the correct spacing between fibers. SLRPs can also regulate apatite formation in mineralized tissues, which are vertebrate innovations, and interact with skeletal growth factors such as TGFs and BMPs (Schaefer and Iozzo 2008; Boskey 2010; Nikitovic *et al*. 2012; Iozzo and Schaefer 2015).

In bony vertebrates, 23 SLRP paralogs have been identified so far and most can be found along 4 different chromosomes (Park *et al*. 2008; Schaefer and Iozzo 2008; Iozzo and Schaefer 2015; Costa *et al*. 2018). They are classically divided into 5 classes based on protein sequence similarity and gene chromosomal localization (Schaefer and Iozzo 2008). Class I contains asporin (Aspn), biglycan (Bgn), decorin (Dcn), and 4 “ECM proteins” (Ecm2, Ecm2L, EcmX, and EcmXL). Class II includes fibromodulin (Fmod), keratocan (Kera), lumican (Lum), lumican-like (LumL), osteomodulin (or osteoadherin, Omd) and prolargin (Prelp). Class III consists of epiphycan (Epyc), opticin (Optc), and osteoglycin (or mimecan, Ogn). Class IV encloses chondroadherin (Chad), chondroadherin-like (ChadL), nyctalopin (Nyx), and tsukushin (Tsku). Class V encompasses podocan (Podn) and podocan-like (PodnL). Nephrocan (Npc) does not belong to any of these classes for structural reasons, despite its classification into SLRPs (Mochida *et al*. 2006; Schaefer and Iozzo 2008; Iozzo and Schaefer 2015; Costa *et al*. 2018). The 16 SLRPs from classes I to III are classified as “canonical” defined by the presence of an extended repeat (called ear repeat) in the LRR C-terminal capping motif (LRRCE) of their protein moieties (Park *et al*. 2008). Despite the expectations of an already diverse canonical SLRP repertoire prior to vertebrate evolution, only a few sequences have been identified in the vertebrate sister group (2 *Ciona* SLRPs with LRRCEs, Park *et al*. 2008). The history of the SLRP family amplification in vertebrates is therefore still uncharacterized, in particular regarding the 2 rounds of whole-genome duplications (2R-event; Ohno 1970) that shaped the evolution of jawed vertebrate genomes, while cyclostome genome evolution shared the first (1R event) and then underwent additional polyploidization events (Nakatani *et al*. 2021; Marlétaz *et al*. 2024).

To assess the evolution of the SLRP gene family in vertebrates we took advantage of high-quality genomic data in the cartilaginous fish lineage (in particular with the small-spotted catshark *Scyliorhinus canicula*) and the cyclostome lineage (including the lampreys *Petromyzon marinus* and *Lethenteron reissneri* and the hagfish *Myxine glutinosa*) together with the cephalochordates *Branchiostoma lanceolatum* and *B. floridae*. We identified the outcome of the 2 rounds of whole-genome duplications (1R and 2R events in gnathostomes; 1R and following events in cyclostomes) on clustered and non-clustered SLRPs. From these, we inferred a hypothetical ancestral state prior to the 1R event and lineage-specific gene losses. Taking advantage of the small-spotted catshark being an amenable organism in the laboratory and with extensive transcriptomic data, we thoroughly characterized SLRP gene expression patterns in this species with a special focus on the processes of differentiation of connective and skeletal tissues. From this, we could infer not only some ancestral features of SLRP expression in jawed vertebrates, but also some derived features of these genes in either the bony or cartilaginous fish lineages.

## Materials and methods

### Protein sequence sampling

SLRP protein sequences were recovered from public databases (NCBI, Genbank) via BLASTP or TBLASTN (Altschul *et al*. 1990) with default parameters of each source database program on 9 sarcopterygians, 7 actinopterygians, and 4 chondrichthyans (the details of the source database for each sequence are given in Supplementary Table 1). The mouse *Mus musculus* and the zebrafish *Danio rerio* sequences were further used to screen the locally assembled transcriptome of the thornback ray *Raja clavata* (Debiais-Thibaud *et al*. 2019). Reciprocal BLASTs were performed to restrain the recovered sequences to actual SLRPs. Seven teleostean and two sarcopterygian sequences for EcmX were recovered via BLAST of the *D. rerio* sequence, to strengthen the resolution in teleosts. *Petromyzon marinus* SLRP sequences (Park *et al*. 2008; Ota *et al*. 2013) were used to screen the *P. marinus* genome (assembly kPetMar1.pri) for additional sequences. These recovered *P. marinus* SLRP sequences were used to screen the *Lethenteron reissneri* and the *Myxine glutinosa* genomes in NCBI (respectively assembly ASM1570882v1 and UKY_Mglu_1.0). The “SLRP” sequences of any *Branchiostoma* species were obtained with BLASTP against the Protein sequence database in NCBI filtered for *Branchiostoma* annotation. The Lingo gene family (including Lingo1, Lingo2, Lingo3, and Lingo4 in jawed vertebrates) were used as an outgroup for the SLRP phylogeny as they contain Leucine-Rich Repeat domains shared with SLRP proteins. Lingo protein sequences were obtained using BLASTP of the Lingo1, Lingo2, Lingo3, and Lingo4 annotated mouse sequences on the *Branchiostoma* and vertebrate species. All sequences used in this study are listed with accession numbers in Supplementary Table 1.

### SLRP phylogeny

The 549 protein sequences from vertebrate and *Branchiostoma* species were aligned with MAFFT v7.453 using the E-INS-i strategy (–genafpair –maxiterate 1000) recommended for sequences with multiple conserved domains and long gaps (Katoh and Standley 2016). The resulting alignment was then filtered to exclude sites containing gaps in more than 95% of the sequences. The final alignment used for subsequent phylogenetic reconstruction included 1,439 amino acids and is available in Supplementary File 1. The phylogeny was then inferred by Maximum Likelihood using IQ-Tree v2.1.3 (Nguyen *et al*. 2015) under the best-fitting model of amino-acid sequence evolution (JTT + R7) as selected using ModelFinder (Kalyaanamoorthy *et al*. 2017) based on the corrected Akaike Information Criterion (AICc). Statistical node support was estimated by performing 1,000 ultrafast bootstrap replicates (“UFBoot”) (Hoang *et al*. 2018) and SH-like approximate likelihood ratio tests based on 1,000 replicates (SH-aLRT; Guindon *et al*. 2010). Branch support (SH-aLRT/UFBoot values) is indicated on the tree files. The full phylogeny is available as a tree file in Supplementary File 2.

### SLRP synteny

SLRP synteny was explored in 7 reference genomes available on NCBI: mouse *M. musculus* (GCF_000001635.27), small-spotted catshark *S. canicula* (GCF_902713615.1), zebrafish *D. rerio* (GCF_000002035.6), elephant shark *Callorhinchus milii* (GCF_000165045.1), sea lamprey *P. marinus* (GCF_010993605.1), Far Eastern brook lamprey *Lethenteron reissneri* (GCF_015708825.1), and hagfish *Myxine glutinosa* (GCF_040869285.1). In addition, the reedfish *Erpetoichthys calabaricus* genome (GCF_900747795.1) was consulted through the UCSC genome browser. Data from *D. rerio* and *C. milii* are shown only when they differ from *E. calabaricus* and *S. canicula*, respectively. *E. calabaricus* was chosen over *D. rerio* as a non-teleost actinopterygian devoid of the 3R (teleost whole-genome duplication event) duplicates.

### SLRP protein domain screening

SLRP protein sequences were screened using LRR finder (Bej *et al*. 2014), EML (cell compartment: extracellular) (Kumar *et al*. 2019), and Sulfinator (Monigatti *et al*. 2002) that predict, respectively, putative positions for Leucine-Rich Repeats (LRRs), glycosaminoglycan and N-glycosylation sites, and tyrosine sulfation sites. Additionally, the proteins were screened for 2 LRRCE motifs: (i) the standard one from (Park *et al*. 2008)⁠ typical of canonical SLRPs; and (ii) a version relaxed in the C-terminal region, using the ScanProsite tool (de Castro *et al*. 2006). The model LRRCE motif was (*PROSITE* syntax):

Standard LRRCE Motif

[LIV]-X(2)-[LVIYFMA]-X-[LIFM]-X(2)-[NH]-X-[ILVF]-X(2)-[VIMFLY]-X(4)-[FIMLV]-C-X(7,20)-[LYIMV]-X(2)-[ILVTMF]-X-[LVMI]-X(2)-N-X-[IVLMAFT]-**X(8,9)-[FYMPVAIS]-X-C**

LRRCE Motif, Relaxed at C-terminal

[LIV]-X(2)-[LVIYFMA]-X-[LIFM]-X(2)-[NH]-X-[ILVF]-X(2)-[VIMFLY]-X(4)-[FIMLV]-C-X(7,20)-[LYIMV]-X(2)-[ILVTMF]-X-[LVMI]-X(2)-N-X-[IVLMAFT]-**X(8,12)-C**

### RT-qPCR

Total RNAs from 22 anterior vertebrae (AV) of *S. canicula* embryos from 5 to 8 cm total length (TL) were isolated with ReliaPrep RNA tissue Miniprep system (Promega) and used for cDNA preparation performed by Superscript II reverse transcription (Invitrogen) with an oligodT primer. Each cDNA was run in triplicate on a 384-well plate for each primer pair by using thermal cycling parameters: 95 °C for 2 min, 95 °C for 10 s, 68 °C for 10 s, 72 °C for 10 s (45 cycles), and an additional step 72 °C for 10 min performed on a LightCycler 480 with the SensiFAST SYBR No-ROX kit (Meridian Bioscience) (qPHD UM2/GenomiX Platform, Montpellier—France). Forward and reverse primers were defined using Primer3Input version 4.1.0 (see Supplementary Table 2). Results were normalized with the expression of 3 reference genes *eef1a*, a*ctin,* and g*apdh* by geometric mean, and data were further analyzed with the LightCycler 480 software 1.5.1. The reference point used was the highest value of ΔCp for a given gene: all expression values of any given gene are all above or equal to 1-fold. Developmental trajectories were plotted on R (v4.04) using the ggplot2 package.

### Embryo collection

Embryos of the small-spotted catshark originated from a Mediterranean population of adult females housed at Observatoire Océanique de Banyuls, France. Embryos were raised in seawater tanks at 16–18 °C and euthanized by an overdose of tricaine (MS222, Sigma) at appropriate stages. Whole embryos were fixed in paraformaldehyde 4% in phosphate-buffered saline solution for 48 h and then stocked in ethanol 100% before the tissue was sampled for cryostat sectioning. Handling of small-spotted catshark embryos followed all institutional, national, and international guidelines [European Communities Council Directive of September 22, 2010 (2010/63/UE)]: no further approval by an ethics committee was necessary as the biological material is embryonic and no live experimental procedures were carried out.

### In situ mRNA hybridization

*In situ* hybridizations were performed on 14-μm-thick cryostat sections of samples cut transversely in the body trunk of fixed 6.5-cm TL embryos, at the level of the pectoral fins. All subsequent procedures were previously described (Leurs *et al*. 2021). Slides were scanned on a Hamamatsu NanoZoomer 2 (Montpellier RIO Imaging facility, INM Optique). Primers designed to generate the DNA matrix used for RNA probe synthesis were defined using Primer3Input version 4.1.0 (see Supplementary Table 2).

## Results

### A conserved SLRP repertoire in vertebrates that diversified in early chordates/early vertebrates

**Fig. 1.**
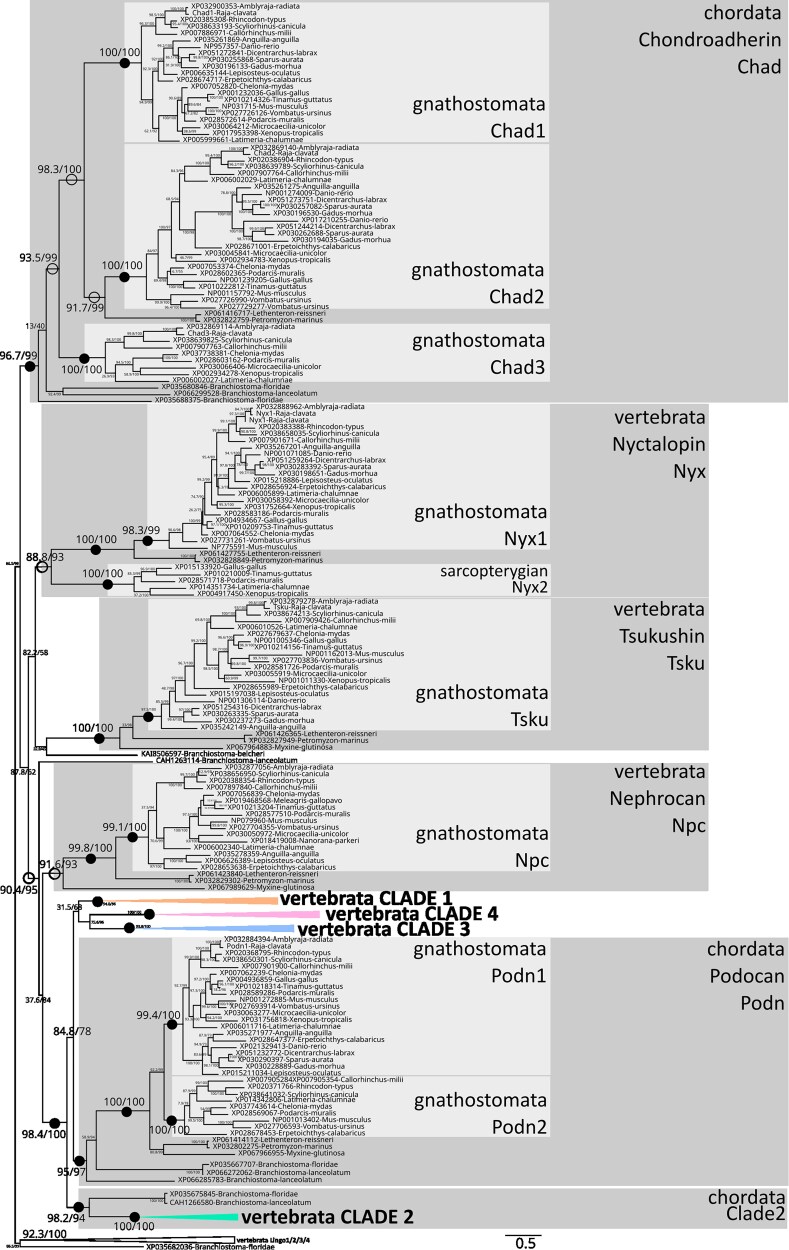
Phylogenetic relationships of SLRP sequences in chordates obtained by maximum likelihood (best fit model JTT + R7; 549 sequences, 1,439 amino-acid positions; full non-collapsed phylogeny given in Supplementary File 2 and Supplementary Fig. 1; non-collapsed Clades 1, 2, 3, and 4 in following Figs. 2, 3, 4, and 5 respectively) and rooted by the vertebrate Lingo1/Lingo2/Lingo3/Lingo4 sequences and their closest amphioxus sequence. Nodes of highlighted clades supported by ultrafast bootstrap and SH-aLRT values both >95 are shown as black dots, or as open circles nodes if values are <95 and >85, SH-aLRT/UFBoot values for internal nodes are shown on the branches. The 4 clades of canonical SLRPs are collapsed and shown in color, non-canonical SLRPs are highlighted in gray.

The complete SLRP phylogeny (Fig. 1 and Supplementary Fig. 1) was rooted using the Lingo protein family as an outgroup, which shares a leucine-rich repeat region with SLRPs but are transmembrane proteins (Homma *et al*. 2009): vertebrate Lingo paralogs grouped with a sister gene annotated as “carboxypeptidase N” in *Branchiostoma floridae*, making this outgroup a chordate-rooted outgroup. This root branched to a first bifurcation separating a chordata Chad clade (non-canonical SLRP, supported by 96.7/99 SH-aLRT/UFBoot values) from the rest of the SLRP proteins. In the Chordata Chad clade were 3 well-supported gnathostomata Chad orthology groups that we named: Chad1 for the classically recognized Chondroadherin paralog, Chad2 for the sometimes identified Chondroadherin-like paralog, and Chad3 for the latter, never identified before, paralog (all supported by 100/100 SH-aLRT/UFBoot). Chad3 sequences were only found in chondrichthyans and in non-amniote sarcopterygians (Supplementary Table 1), and a single lamprey sequence was 1:1 orthologous to Chad2. These phylogenetic relationships could have suggested an origin of 3 gnathostome paralogs from the 2 rounds of whole-genome duplication. However, *chad2* and *chad3* were found to be tandemly arranged genes in the elephant shark *C. milii* and the small-spotted catshark (on loci NW_006890314.1:268,311–282,341 and chr23: 22,986,334–23,090,131 respectively), invalidating this hypothesis.

Sister to the chordata Chad clade, low support of branches resolved in a trifurcation joining the vertebrata Tsku clade (100/100 SH-aLRT/UFBoot), the vertebrata Nyx clade (88.8/93), and the remaining SLRPs that appear monophyletic with intermediate support values (90.4/95). The Nyx clade was made of 2 gnathostomata paralogs (herein named Nyx1 and Nyx2, respectively 98.3/99 and 100/100) and 2 lamprey sequences. The vertebrate Tsku clade included one copy for each species.

In the remaining part of the tree, the gnathostomata Npc clade (one gene for each species, 91.6/93) was sister to a clade made of a multifurcation of 5 well-supported groups of orthology: (i) the chordata Podn clade (95/97) with 2 gnathostome paralogs herein named Podn1 and Podn2 (99.4/100 and 100/100); (ii) the chordata clade herein named Clade 2 (100/100); (iii) the vertebrata Clade 1 (94.6/94); (iv) the vertebrata Clade 3 (99.8/100); (v) and the vertebrata Clade 4 (100/100).

Amphioxus sequences robustly grouped with vertebrate sequences in the Podn, Clade 2, and Chad chordata clades (Fig. 1), placing the origin of each of these clades earlier than the divergence of the cephalochordate lineage from the other chordate lineages. Although less robust in our results (90.4/95 SH-aLRT/UFBoot), the grouping of (chordate Clade 2; chordate Podn; and vertebrate Clades 1, 3, and 4) rather support the same age for the origin of the Tsku and Nyx clades, although only identified in vertebrate species. In the elephant shark *C. milii* and the small-spotted catshark genomes*, npc*, *nyx1*, *podn1*, *podn2,* and *tsku* were all located as single SLRPs on independent loci. However, in *Branchiostoma lanceolatum*, the Podn- and Clade 2-associated genes are located 56 kb apart on chromosome 5, opening the hypothesis of an initial tandem duplication at the origin of the Podn and Clade 2 ancestral genes.

### Canonical SLRP paralogs were amplified both with the gnathostome 2R events and local tandem duplications

Clade 2 includes vertebrate and amphioxus sequences, but Clades 1, 3, and 4 only include vertebrate sequences (Fig. 1), placing the origin of these 3 later clades at an undetermined timing located between the time of divergence of cephalochordate from other chordates and the time of cyclostome/gnathostome divergence. In the following, we focus on the gnathostome groups of orthology and their relationship to cyclostome SLRPs (whose sequences we named SLRP 1 to SLRP13, with numbers randomly distributed) so as to better identify events of duplication associated with the 1R + 2R events.

**Fig. 2.**
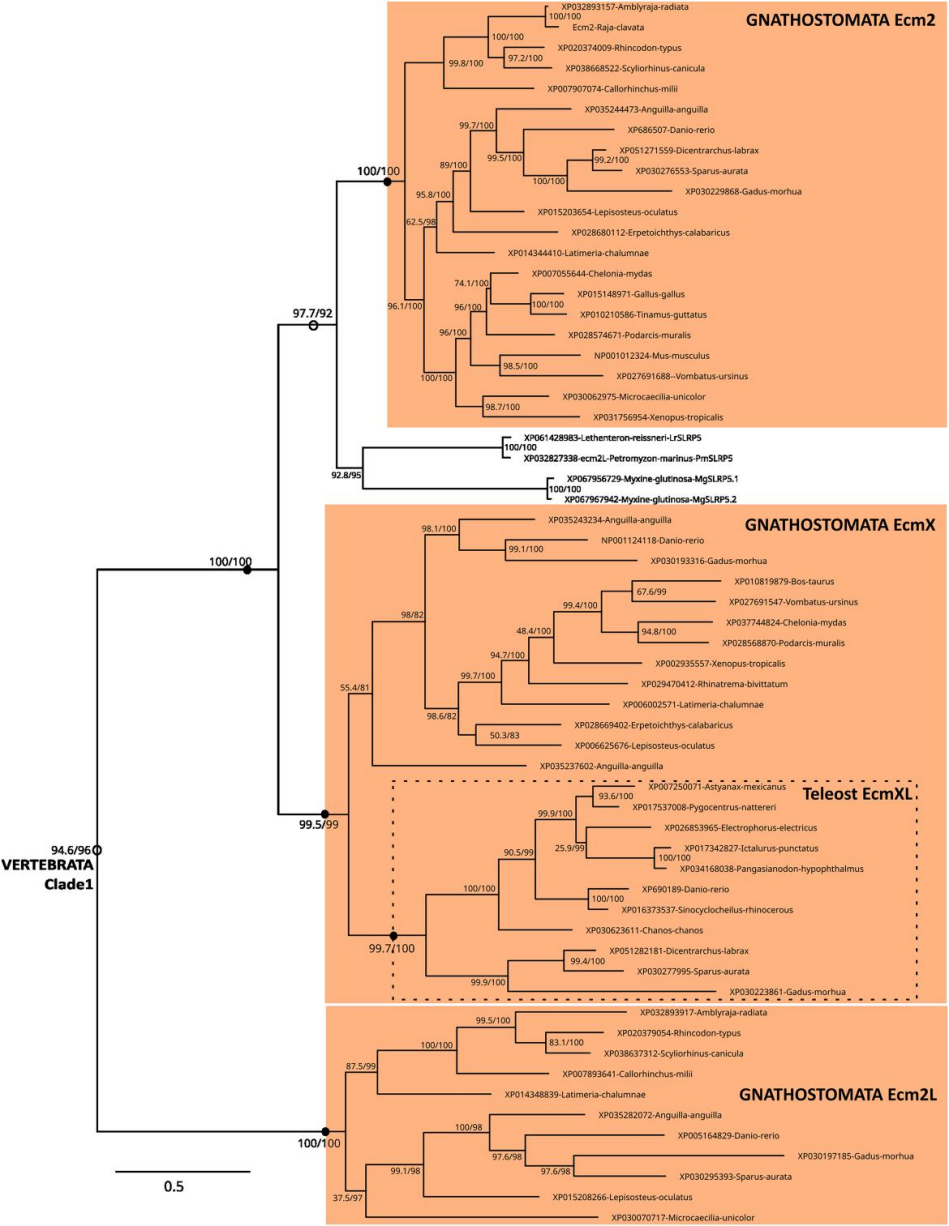
Detail of internal nodes within the vertebrate Clade 1 as defined in Fig. 1. Nodes of highlighted clades supported by ultrafast bootstrap and SH-aLRT values both >95 are shown as black dots, or as open circles nodes if values are <95 and >85, SH-aLRT/UFBoot values for internal nodes are shown on the branches. Dotted box locates teleost-specific duplicates.

Within Clade 1, 3 gnathostome paralogs were identified as Ecm2, EcmX, and Ecm2L (Fig. 2) with Ecm2L lost in most tetrapods and EcmX lost in chondrichthyans and birds. Ecm2 was the sister clade to EcmX with the placement of cyclostome SLRP5 as sister group of Ecm2 (SH-aLRT/UFBoot: 97.7/92; Fig. 2). Within the EcmX clade, a teleost-only clade (99.7/100) branched out of all other bony fish sequences, but low internal node support prevented from supporting either of 2 possible scenarios: this clade was previously identified as EcmX and considered another Ecm2 related gnathostome paralog (Costa *et al*. 2018), or it may be the product of the teleost-specific third whole-genome duplication identified as the 3R event ⁠(Jaillon *et al*. 2004; Kasahara *et al*. 2007).

**Fig. 3.**
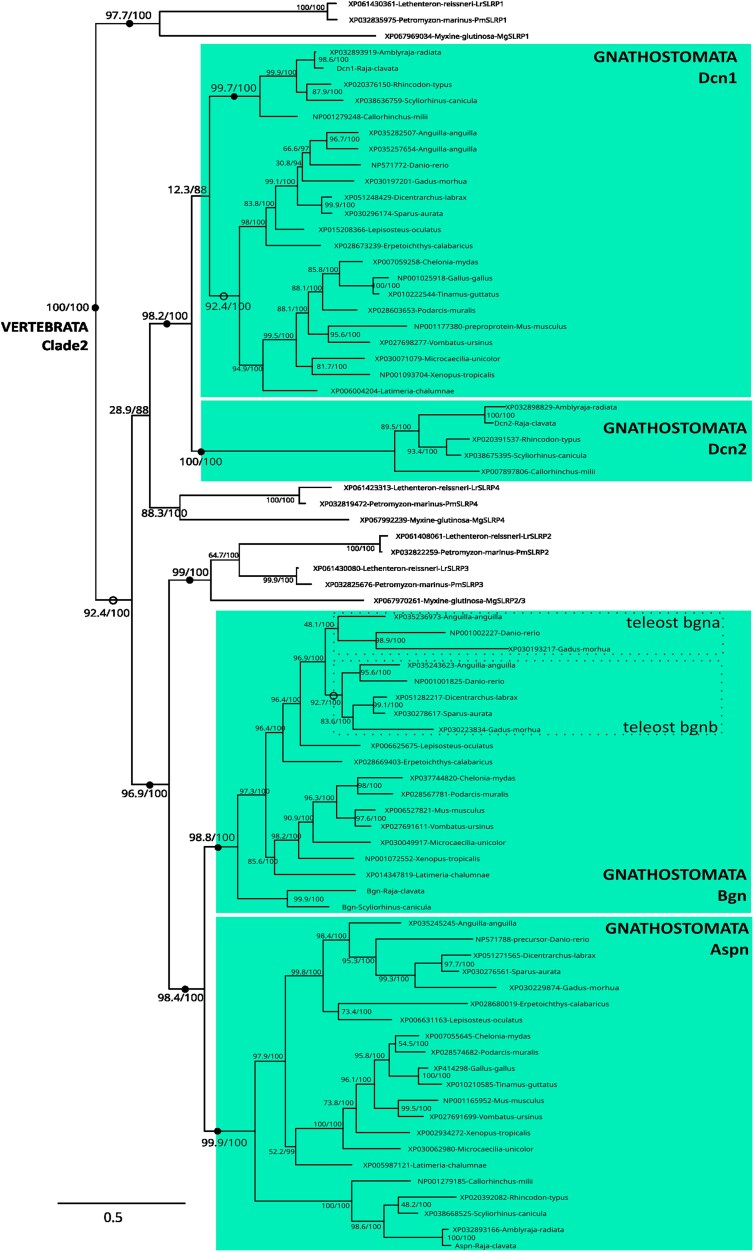
Detail of internal nodes within the vertebrate Clade 2 as defined in Fig. 1. Nodes of highlighted clades supported by ultrafast bootstrap and SH-aLRT values both >95 are shown as black dots, or as open circles nodes if values are <95 and >85, SH-aLRT/UFBoot values for internal nodes are shown on the branches. Dotted boxes locate teleost-specific duplicates.

Within Clade 2 (Fig. 3) gnathostome Bgn and Aspn were sister clades and teleost 3R duplicates were only conserved for Bgn (*bgna* and *bgnb*). Their cyclostome sister clade included: 2 paralogs in lampreys (herein identified as SLRP2 and SLRP3) and one myxine paralog (SLRP2/SLRP3). The gnathostome Dcn clade (98.2/100) included one clade with osteichthyans and chondrichthyan sequences (Dcn1), and a second clade with only chondrichthyan sequences (Dcn2), despite poor support for the Dcn1 clade (see next section on synteny for a robust argument to support with group of orthology). The cyclostome SLRP1 and SLRP4 clades were placed as the outgroup of all other, or as part of the (Dcn1, Dcn2, Aspn, and Bgn) clade, respectively.

**Fig. 4.**
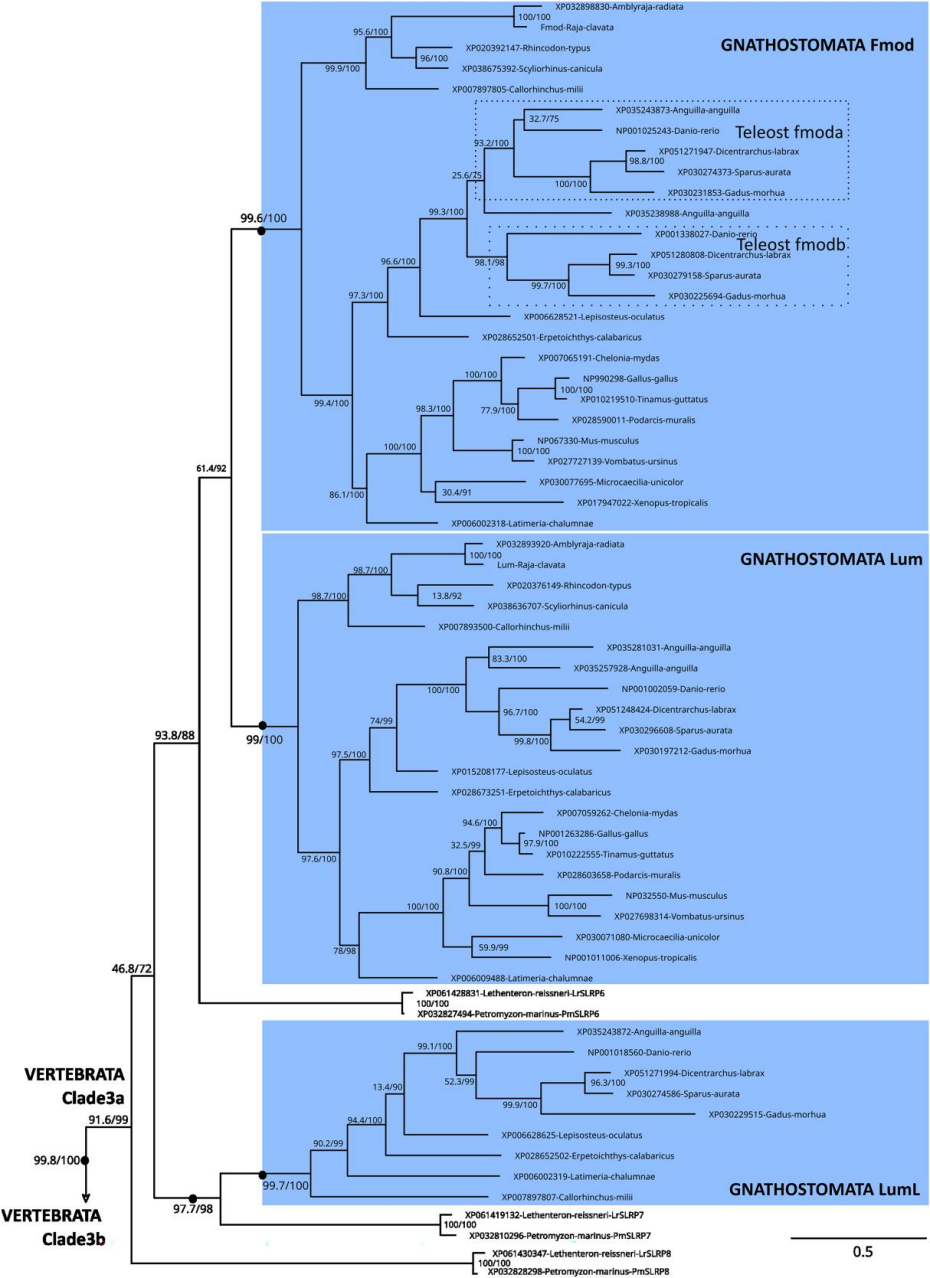
Detail of internal nodes within the vertebrate Clade 3 as defined in Fig. 1: Clade 3a. Nodes of highlighted clades supported by ultrafast bootstrap and SH-aLRT values both >95 are shown as black dots, SH-aLRT/UFBoot values for internal nodes are shown on the branches. Dotted boxes locate teleost-specific duplicates.

**Fig. 5.**
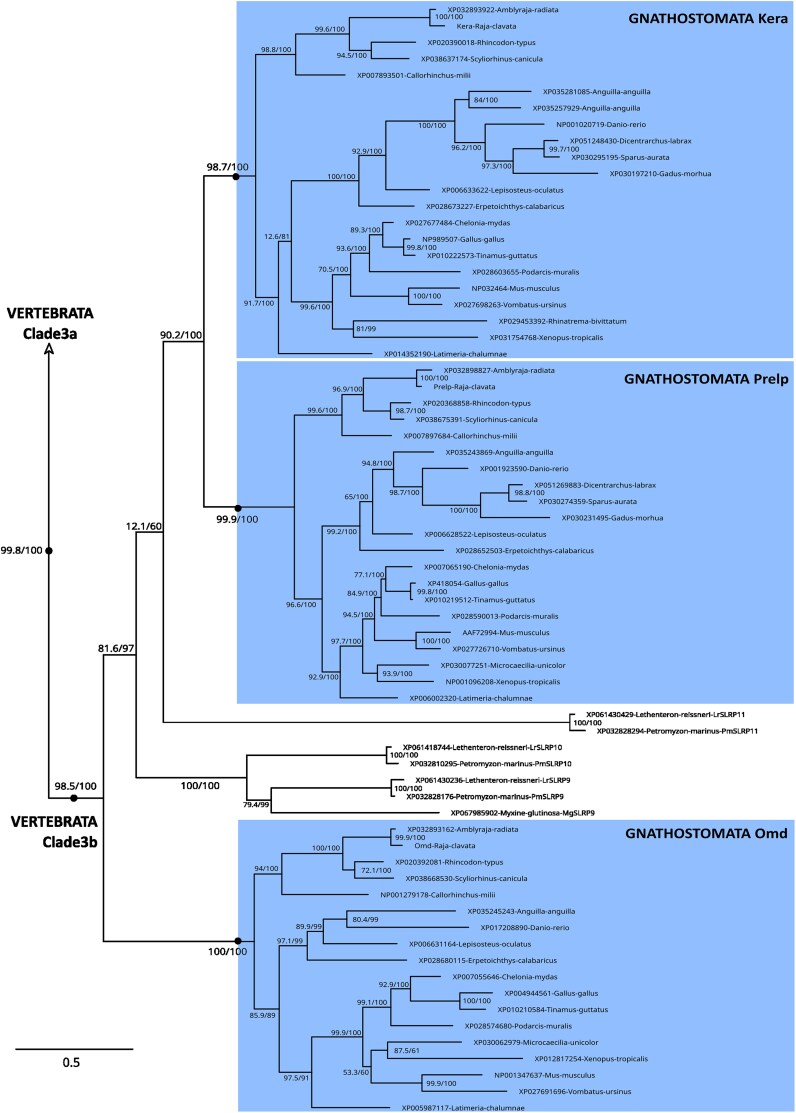
Detail of internal nodes within the vertebrate Clade 3 as defined in Fig. 1: Clade 3b. Nodes of highlighted clades supported by ultrafast bootstrap and SH-aLRT values both >95 are shown as black dots, SH-aLRT/UFBoot values for internal nodes are shown on the branches.

We displayed the phylogeny of Clade 3 by illustrating the 2 major clades that were identified into Clade 3: the gnathostome Lum, Fmod, and LumL groups and 3 cyclostome (lamprey) sequences constituted Clade 3a (SLRP6, 7, and 8; Fig. 4), sister to Clade 3b and made of the gnathostome Omd, Kera and Prelp groups along with another 3 cyclostome sequences (SLRP9, SLRP10, and SLRP11; Fig. 5). In Clade 3a, teleost 3R paralogs were identified only in the Fmod clade, while LumL was identified only in elephant shark, the coelacanth and actinopterygian species, so inferred to be secondarily lost in elasmobranchs and in tetrapods. LumL was the only paralog for which a 1:1 orthology relationship with the cyclostome SLRP7 was identified. Lamprey paralogs SLRP6 and SLRP8 could not be robustly identified as sister to either of the gnathostome paralogs (Fig. 4).

In Clade 3b, all gnathostome lineages displayed the presence of the Kera, Prelp, and Omd paralogs, with only one copy of each in teleost species, despite the 3R event (Fig. 5). No 1:1 orthology relationship could be identified between the gnathostome clades and the cyclostome SLRP 9, SLRP10 or SLRP11.

**Fig. 6.**
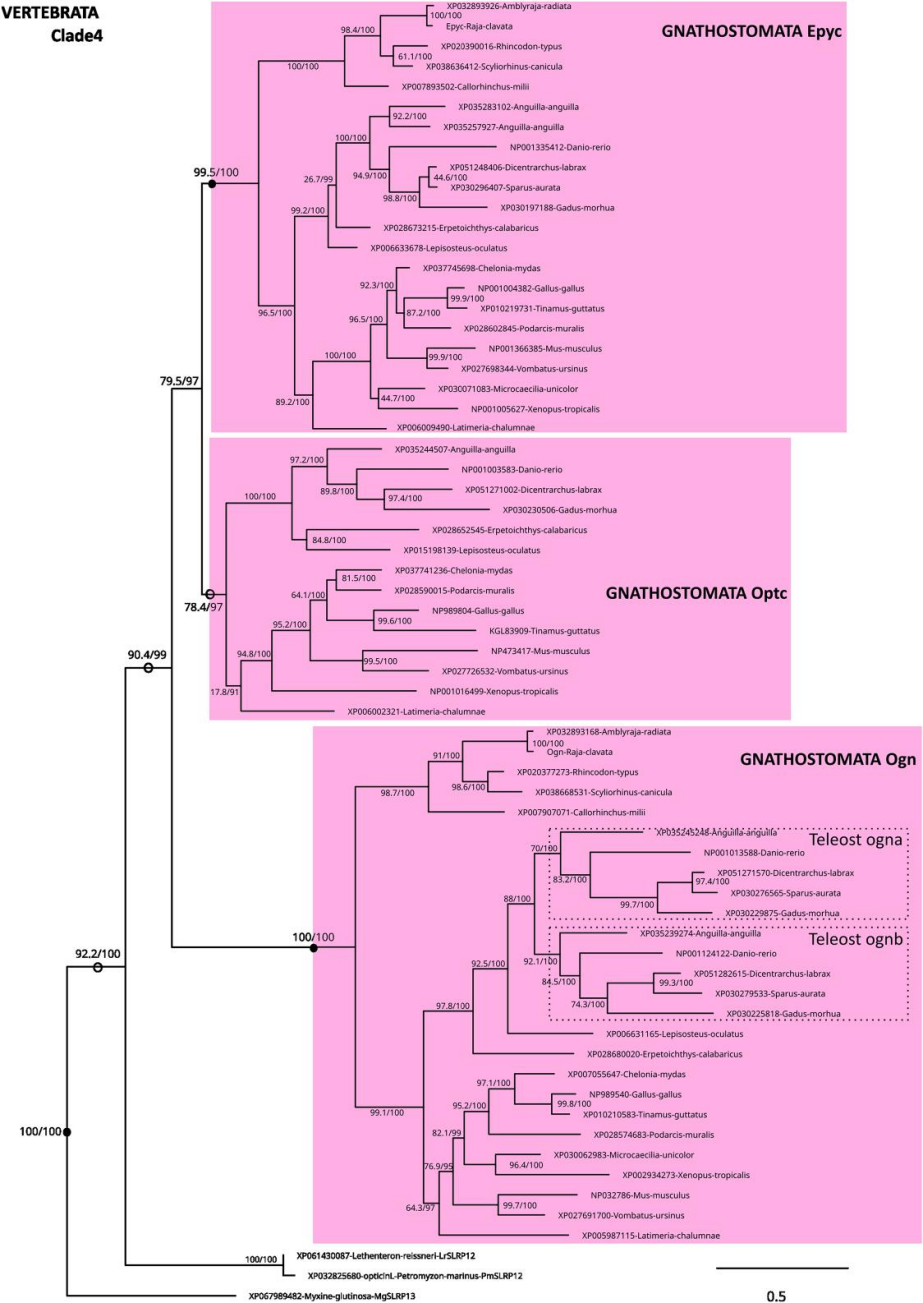
Detail of internal nodes within the vertebrate Clade 4 as defined in Fig. 1. Nodes of highlighted clades supported by ultrafast bootstrap and SH-aLRT values both >95 are shown as black dots, or as open circles nodes if values are <95 and >85, SH-aLRT/UFBoot values for internal nodes are shown on the branches. Dotted boxes locate teleost-specific duplicates.

Within Clade 4, the gnathostome Ogn and Epyc clade were well supported, but the Optc group had lower support (78.4/97) together with the sister relationship between Optc and Epyc (79.5/97; Fig. 6). The Optc paralog was lost in chondrichthyans. Teleost-specific duplication was partially conserved for ogn (*ogna* and *ognb*) as previously shown (Costa *et al*. 2018). The cyclostome sequences branched outside of the whole clade of gnathostome sequences, and as the hagfish sequence branched even outside of all others, we decided to name it SLRP13, when the lamprey sequences were named SLRP12.

### Genomic organization of the vertebrate canonical SLRPs

**Fig. 7.**
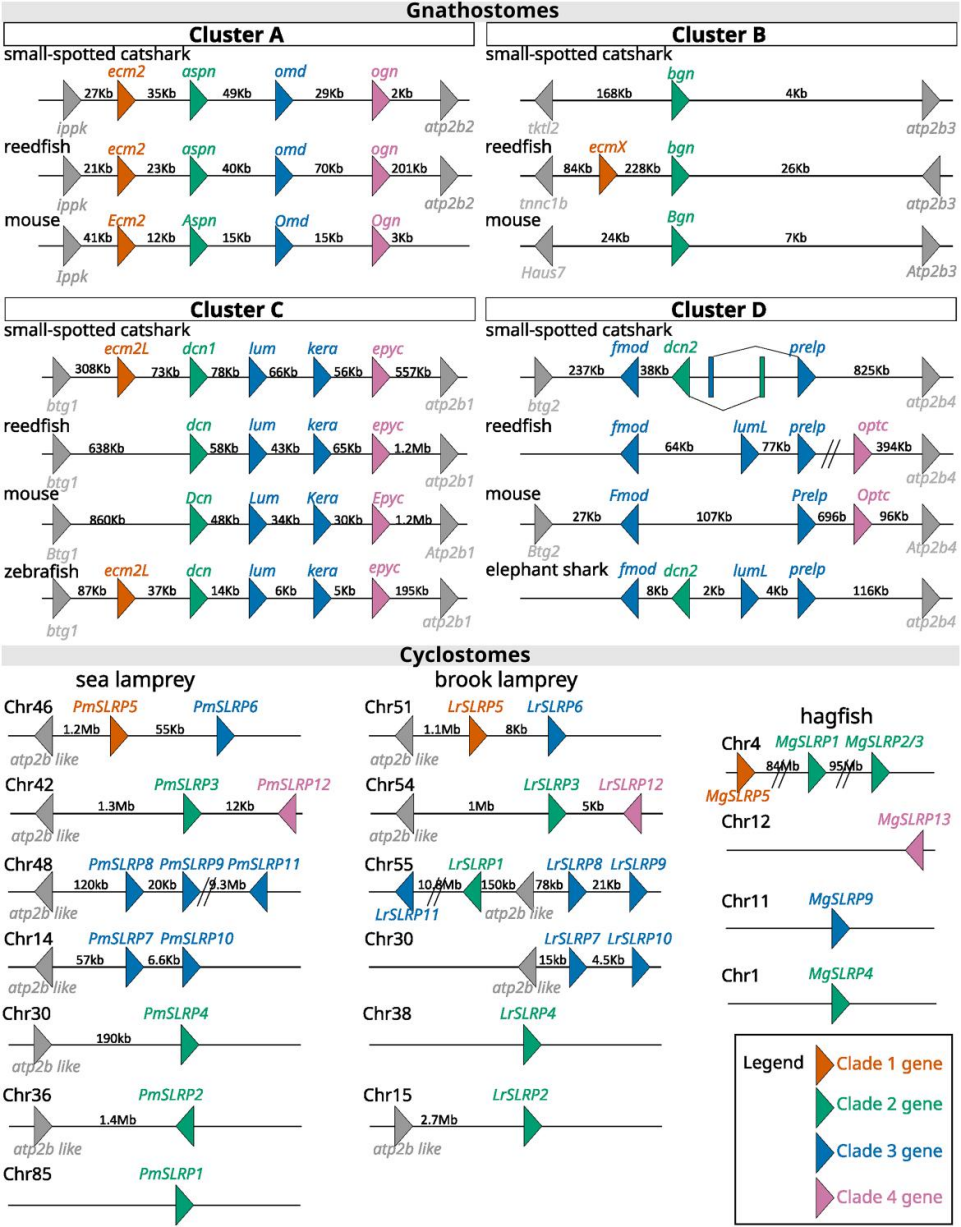
Genomic organization of canonical SLRP gene clusters in selected vertebrate genomes. Distance between genes is indicated but not represented to scale. Gene names are similar to Fig. 1 (for gene accession numbers, see Supplementary Table 1).

In gnathostome species, canonical SLRP genes were clustered along 4 conserved genomic loci (Fig. 7, and Supplementary Table 1 for detailed genomic location). These gene clusters will be referred to as gene clusters A, B, C, and D. Genes found on cluster A were *ecm2*, *aspn*, *omd,* and *ogn*; on cluster B: *ecmX* (illustrated here with the reedfish *E. calabaricus*) and *bgn*; on cluster C: *ecm2L* (in the catshark and zebrafish), *dcn1*, *lum*, *kera*, and *epyc*; on cluster D: *fmod*, *dcn2* (only in cartilaginous fishes), *lumL* (in the reedfish and elephant shark), *prelp*, and *optc* (only found in bony fishes). For each cluster, at least one neighbor gene was conserved in synteny between the compared genomes, supporting orthology between compared loci (Fig. 7). We oriented all clusters so that the first position along the gene cluster was occupied by Clade 1 genes (*ecm2*, *ecmX*, *ecm2L*; absent on cluster D) and/or so that the last position of the gene cluster was occupied by Clade 4 genes (*ogn*, *epyc*, and *optc*) (absent on cluster B). Clade 2 and 3 genes showed less conserved positions on cluster D (Fig. 7). Clade 2 genes (*aspn*, *bgn*, and *dcn1*) were positioned on the second locus in clusters A, B, and C but *dcn2* was positioned between *fmod* and *lumL* on cluster D (Fig. 7). The location of gnathostome *dcn1* genes on one orthologous locus along gene cluster C in all gnathostomes led us to support the monophyly of gnathostome Dcn1 clade despite its low support in the phylogeny (Fig. 3). No Clade 3 gene was found on cluster B: one paralog was identified on cluster A (*omd*), 2 paralogs on cluster C (*lum* and *kera*) and a maximum of 3 paralogs on cluster D (*fmod*, *lumL,* and *prelp*).

In the lamprey genomes, 4 SLRP gene tandems were identified (Fig. 7). *SLRP5* shared synteny with *SLRP6* (Clade 1 and 3 genes, respectively), and *SLRP3* shared synteny with *SLRP12* (Clade 2 and 4 genes, respectively). *SLRP8*, *SLRP9,* and *SLRP11* all belonged to Clade 3 and were all found on the same chromosome, but *SLRP11* was megabases away from the 2 other clustered sequences, making this shared synteny a potential result of secondary chromosomal rearrangements. In the brook lamprey, *LrSLRP1* was also found in the same chromosomal region, but again, more than 200 kb away from its closest *LrSLRP8* gene. Finally, *SLRP7* was found with *SLRP10* (both Clade 3 genes). Shared synteny with a sequence coding for a member of the *Atp2b* gene family was verified on most of the loci where a lamprey *SLRP* gene was identified (Fig. 7), showing a greater number of homologous loci linking canonical SLRPs with an ATP2B-related gene in cyclostomes (a minimum of 6) than in gnathostome (a maximum of 4). No such clustering of SLRP genes could be identified in the hagfish genome (Fig. 7) as only *SLRP1*, *SLRP2/3,* and *SLRP5* were found on the same chromosome but megabases away one from another.

### Conservation of SLRP protein domains in vertebrates

We screened specific SLRP protein structural data, such as LRR number and length and putative N-glycosylation or glycosaminoglycan attachment sites (see Supplementary Table 3). We found a small degree of variation in the number of LRRs in the small-spotted catshark proteins which often lacked 1–2 LRRs as compared to mammal sequences (Matsushima *et al*. 2021). Within the small-spotted catshark canonical SLRPs, we found a standard LRRCE motif in Aspn, Ecm2, Dcn1, Dcn2, Lum, Ogn, and Epyc. In the remaining sequences, only a screening with the C-terminal unconstrained LRRCE yielded hits. In addition to the LRR and LRRCE motifs, protein alignment showed good conservation of the cysteine-rich motifs on the N-terminal capping region of all small-spotted catshark SLRPs (Low *et al*. 2021). Several small-spotted catshark SLRP sequences showed high similarity in terms of the number and position of putative N-glycosylation and glycosaminoglycan attachment sites (e.g. Clade 3 SLRPs except Omd) compared to described bony fish orthologs. Twelve lamprey SLRPs belonged to the 4 clades where gnathostome canonical sequences were found. In both lampreys, 10 sequences out of these 12 also displayed a standard LRRCE motif (SLRP1–3, SLRP5–8, SLRP10, and SLRP12), while in the hagfish, 5 out of 6 canonical SLRP displayed a standard or relaxed LRRCE motif (SLRP1, SLRP2/3, SLRP5, SLRP9, and SLRP13). No LRRCE motif was identified in any of the *Branchiostoma* sequences but displayed 63 LRR domains.

### Tissue- and embryonic stage–specific expression of SLRP genes in the small-spotted catshark

Gene expression levels were extracted from transcriptomic data published in Mayeur *et al*. (2024). These transcriptomic data were acquired in a variety of small-spotted catshark embryonic stages and adult tissues and were compared through TPM values (a proxy for expression quantification in a given sample) and *Z*-score values (a proxy to quantify overexpression bias toward one given sample), both summarized in Table 1 (and see Supplementary Tables 4 and 5). Some SLRP transcripts displayed very low TPM values across all sampled tissues (*dcn2*, *kera*, *chad3*, *npc*, *nyx*, *podn1,* and *podn2*, mean TPM < 15), while others reached high expression levels in some tissues (e.g. *chad1* in vertebrae > 2,700 TPM). In most sampled tissues, *bgn* displayed high levels of expression (TPM > 50). Expression of many SLRPs was biased (*Z*-score > 1) toward endoskeletal tissues (the Meckel's cartilage, vertebrae, and chondrocranium samples) and/or exoskeletal tissues (dental lamina), and some were exclusively enriched in the endoskeletal system: *chad1*, *chad2*, *epyc, fmod,* and *lum*.

**Table 1.** Selection of transcriptomic data (TPM values) to characterize SLRP gene expression profiles in adult skeletal tissues, sensory organs, and embryonic stages of the small-spotted catshark, data extracted from Mayeur *et al*. (2024).

		Meckel's cartilage	Vertebrae	Chondro-cranium	Skin denticles	Dental lamina	Eye	AOL	E-St-12	E-St-22	E-St-24	E-St-26	E-St-30	E-St-31
Clade 1	*ecm2*	**63.0**	**64.9**	**46.7**	18.7	32.6	24.4	**45.6**	7.5	10.0	13.4	13.2	26.4	19.9
	*ecm2L*	1.0	0.9	1.3	0.9	1.3	1.3	1.0	1.0	**141.5**	**182.2**	**149.2**	4.3	1.8
Clade 2	*aspn*	**129.2**	**130.9**	54.2	30.0	**130.6**	33.5	26.5	1.2	1.5	2.6	2.4	33.0	61.6
	*bgn*	388.0	**711.6**	**550.2**	208.0	170.1	284.1	**1048.8**	1.0	38.6	119.7	63.1	134.3	121.1
	*dcn1*	**93.0**	**244.5**	29.9	22.9	33.0	4.1	7.4	3.5	2.2	6.2	8.6	50.6	**72.7**
	*dcn2*	2.1	2.5	1.0	0.5	0.5	4.5	0.5	0.2	0.5	0.5	0.9	**5.9**	**11.0**
Clade 3	*fmod*	**144.7**	**305.2**	56.1	6.4	7.6	7.1	6.5	6.0	6.3	12.1	9.9	16.8	8.8
	*kera*	4.5	4.3	3.6	4.3	5.2	4.8	3.7	2.9	5.3	9.0	15.6	**48.7**	**31.1**
	*lum*	**688.7**	**825.9**	259.9	93.6	211.3	51.2	268.6	1.0	16.1	8.6	24.4	119.1	189.0
	*omd*	33.4	31.1	26.3	21.6	32.9	17.8	22.8	14.8	13.8	17.3	16.2	**93.3**	**133.3**
	*prelp*	**118.5**	**222.6**	**124.0**	59.6	48.5	71.6	**103.8**	9.5	17.1	18.9	19.5	51.3	44.7
Clade 4	*epyc*	199.1	**1939.3**	46.1	1.9	2.6	9.0	2.9	1.3	42.6	39.4	49.2	43.5	16.1
	*ogn*	**152.1**	**246.9**	**136.9**	58.7	39.5	55.4	70.8	1.3	1.6	2.8	2.7	91.8	**129.3**
Non-canonical	*chad1*	**1325.9**	**2715.1**	**704.3**	12.0	4.0	57.7	9.8	0.5	0.4	0.9	3.2	37.9	21.3
	*chad2*	**234.7**	**152.7**	**90.1**	0.5	1.8	9.5	3.3	1.2	3.9	4.2	6.9	17.4	10.0
	*chad3*	9.5	7.4	15.2	9.9	6.6	13.9	11.0	1.8	6.1	9.4	8.7	10.6	11.5
	*npc*	0.1	0.1	0.1	0.0	0.0	0.0	0.1	0.1	**0.9**	**1.1**	**1.7**	0.1	0.1
	*nyx*	2.7	2.7	2.6	2.0	2.8	**5.7**	2.2	1.3	2.1	**3.1**	2.2	**3.3**	2.6
	*podn1*	**9.5**	**13.6**	**17.4**	1.1	1.7	**9.0**	8.2	0.3	0.7	1.7	3.4	**10.3**	**8.9**
	*podn2*	**40.7**	**32.0**	**24.3**	14.0	16.2	18.0	10.1	9.6	11.1	13.9	11.8	12.3	10.3
	*tsku*	21.6	15.0	18.6	33.4	**50.3**	36.1	11.9	22.1	23.0	15.3	26.0	23.0	20.2

Samples with both *Z*-score > 1 and TPM > 50 are bold and underlined. Samples with *Z*-score > 1 but TPM < 50 are bold. AOL: ampullae of Lorenzini; E-St-X: embryo stage x. See the complete tables with the 31 adult tissues/embryo stage TPM and *Z*-score values in Supplementary Tables 4 and 5.

Several SLRPs from different clusters and different clades had expression biased towards the ampullae of Lorenzini (AOL) (*ecm2*, *bgn*, and *prelp*, Table 1) or the eye (*nyx* and *podn1)*, making these sensory organs another important site of SLRP expression. Biased expression was also found for *ogn* in the esophagus (Supplementary Tables 4 and 5). Compared to the other SLRPs, *tsku* displayed a more divergent expression pattern being the only one enriched in the liver, spiral intestine and the uterus but not in endoskeletal tissues (Supplementary Tables 4 and 5). Finally, *ecm2L* was exclusively enriched in early embryonic stages. Most SLRPs also showed strong and/or biased expression in late embryonic stages (stages 30 and 31), up to an exclusive expression of *dcn2*, *kera,* and *omd* for these stages, when the skeleton is known to engage in cell differentiation [Table 1 and (Enault *et al*. 2016; Berio *et al*. 2021)]. Based on parallel higher enrichment in the skeletal tissues and in late embryonic stages, we further controlled for SLRP expression during skeletal development in later small-spotted catshark embryonic stages.

### Cell-specific expression of SLRPs in developing skeletal tissues of the small-spotted catshark

**Fig. 8.**
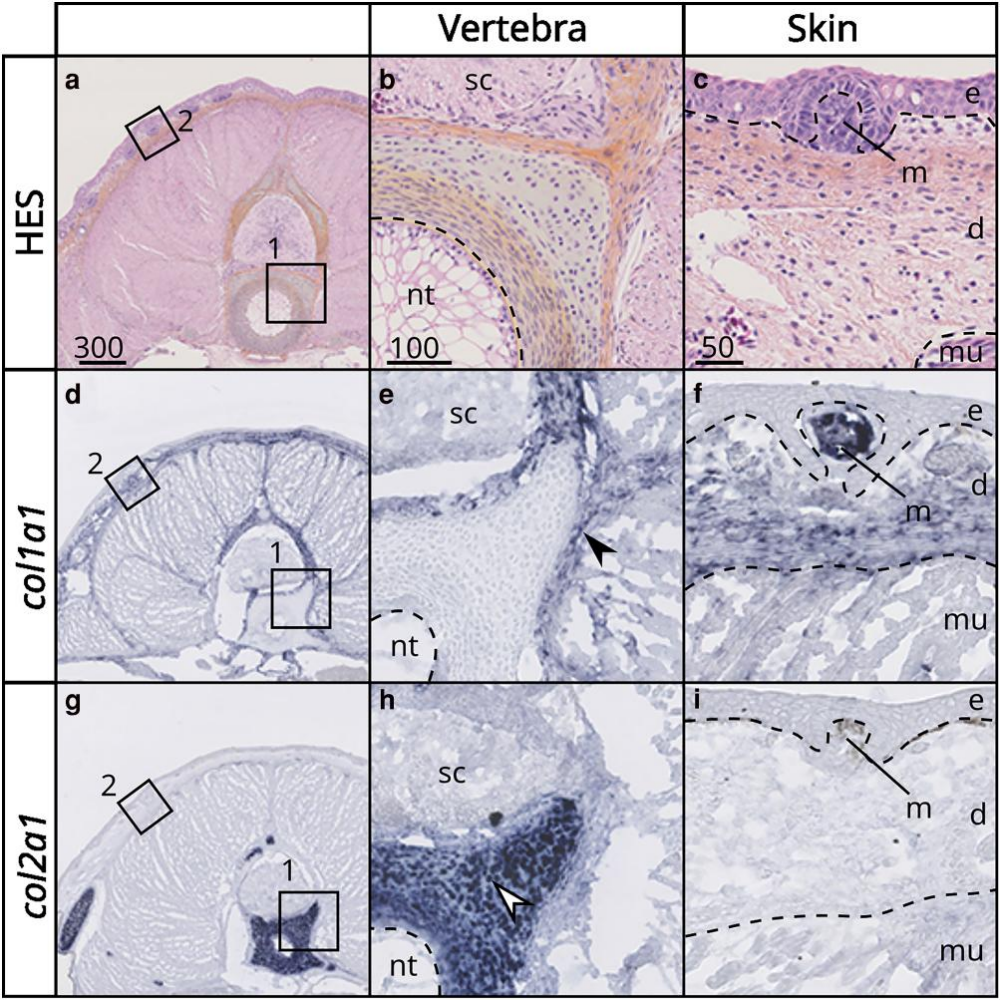
Histology and gene expression in anterior cross sections of 6.5 cm TL *S. canicula* embryo. a–c) HES staining, d–f) *col1a1* in situ hybridization; g–i) *col2a1* in situ hybridization. Close-up on b, e, h) vertebral tissues and c, f, i) skin layers. Legends: d: dermis; e: epidermis; m: mesenchyme of a scale; mu: muscle; nt: notochord; sc: spinal cord. Black arrowheads indicate expression in the perichondrium; white arrowheads indicate expression in chondrocytes. Dotted lines mark separations between tissues. Scale bars are in micrometers and given at the top of each column.

We tested the timing and location dynamics of genes expressed in endoskeletal tissues (vertebrae) first by relative qPCR measurement (Supplementary Fig. 2) and then by *in situ* hybridization on embryonic tissues. The results of qPCR amplification showed that several SLRPs were lowly expressed or even could not be amplified in our samples of embryonic vertebrae: *kera*, *ecm2L*, *dcn2*, *chad3*, *podn1*, *podn2*, *npc*, *nyx,* and *tsku*. These genes were therefore not selected for further analysis by *in situ* hybridization. The relative qPCR data showed that most of the SLRPs expressed at that stage were downregulated over the course of tissue differentiation (Supplementary Fig. 2) except for 4 of them (out of 18 genes): *omd*, *lum*, *fmod* and *podn1* (Supplementary Fig. 2). In 5- to 8-cm TL small-spotted catshark embryos, skeletal tissues develop from poorly differentiated cell populations to a variety of differentiated tissues including: non-mineralized and mineralized cartilage and perichondrium, mineralized fibrous sheath of the notochord and non-mineralized notochord [Fig. 8, a–c and (Enault *et al*. 2016; Berio *et al*. 2021)], skin denticles develop as the dermis and epidermis differentiate, muscle tissue differentiates (Fig. 8c). To better identify the cell-specific gene expression patterns of SLRPs, we therefore used *in situ* hybridization on sections of 6.5 cm TL embryos for a selected set of genes (based upon TPM values (mean endoskeletal tissue TPM > 30, Table 1) and positive results of qPCR amplification).

**Fig. 9.**
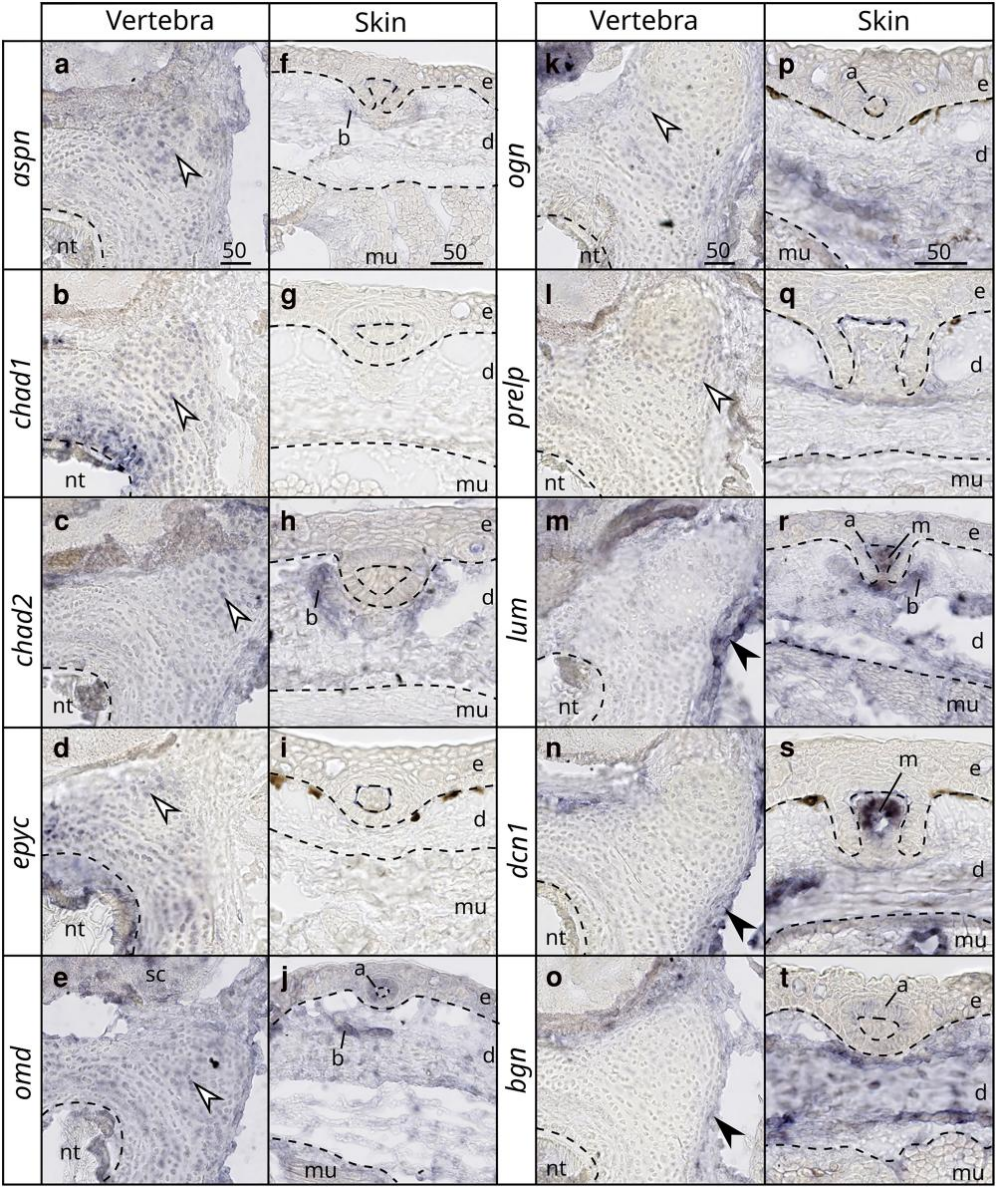
mRNA *in situ* hybridizations of selected SLRPs in vertebral tissues (a–e and k–o) and skin layers (f–j and p–t) in 6.5 cm TL *S. canicula* embryo. Legends: a: ameloblast; b: scale base and legends as in Fig. 8. Scale bars are in micrometers and given on the top panel of each column.

Expression patterns of other SLRPs followed 2 main patterns and were compared to either *col2a1* or *col1a1* expression (Fig. 8) that were assigned to either chondrocyte or perichondrial cells, respectively. The *aspn*, *chad1*, *chad2*, *epyc*, *omd*, *ogn,* and *prelp* genes were expressed by chondrocytes (Fig. 9, a–e and k, l), while *bgn*, *dcn1,* and *lum* displayed perichondrial cell expression (Fig. 9, m–o). Additional expression sites were observed: in chordocytes of the notochord for both *chad1* and *epyc* and in neural cells of the spinal cord for *omd*. In developing scales, ameloblastic expression was detected for *bgn*, *lum*, *omd,* and *ogn* (Fig. 9, j, p, r and t), while mesenchymal (odontoblastic) expression was observed for *dcn1* and *lum* (Fig. 9, r and s). Additionally, expression was detected in the mesenchyme of scale roots for *aspn*, *chad2*, *lum,* and *omd* (Fig. 9, f, h, j and r). Expression in the dermal cells was detected for *bgn*, *dcn1*, *lum*, *omd,* and *ogn* (Fig. 9, j, p and r–t). Finally, muscle expression was detected for *dcn1*, *lum*, *omd,* and *bgn* (Fig. 9, j and r–t). No expression could be detected by *in situ* hybridization for *ecm2* and *fmod*.

## Discussion

In this study, we recovered previously described gnathostome orthology groups, resolved the phylogenetic relationships among canonical SLRPs within each Clade 1, 2, 3, and 4, and identified several new gnathostome paralogs of the SLRP family: one canonical SLRP (Dcn2, only conserved in chondrichthyans); and 3 non-canonical SLRPs that we named Nyx2, Podn2, and Chad3.

By integrating amphioxus sequences in the phylogeny, we can infer that all non-canonical SLRP clades evolved before the divergence between vertebrates and cephalochordates, despite the fact that some of them were not conserved in extant cephalochordates (Fig. 1). Only one amphioxus sequence showed orthology relationships with canonical SLRP sequences, all grouped into the chordate Clade 2, also supporting the evolution of Clade 2 in an early chordate ancestor. In the genome of the amphioxus *Branchiostoma lanceolatum,* the genes identified in the Podn and Clade 2 groups were genes arranged in tandem on chromosome 5, supporting a single zone of tandem duplications that evolved into the Podn and Clade 1, 2, 3, and 4 genes. In our phylogeny, cephalochordate/vertebrate orthology relationships could not be inferred for Clades 1, 3, and 4: these tandem duplications may therefore have occurred later, but still before the last common ancestor of extant cyclostome and gnathostome.

### Evolutionary scenario for canonical SLRP expansion in vertebrates

**Fig. 10.**
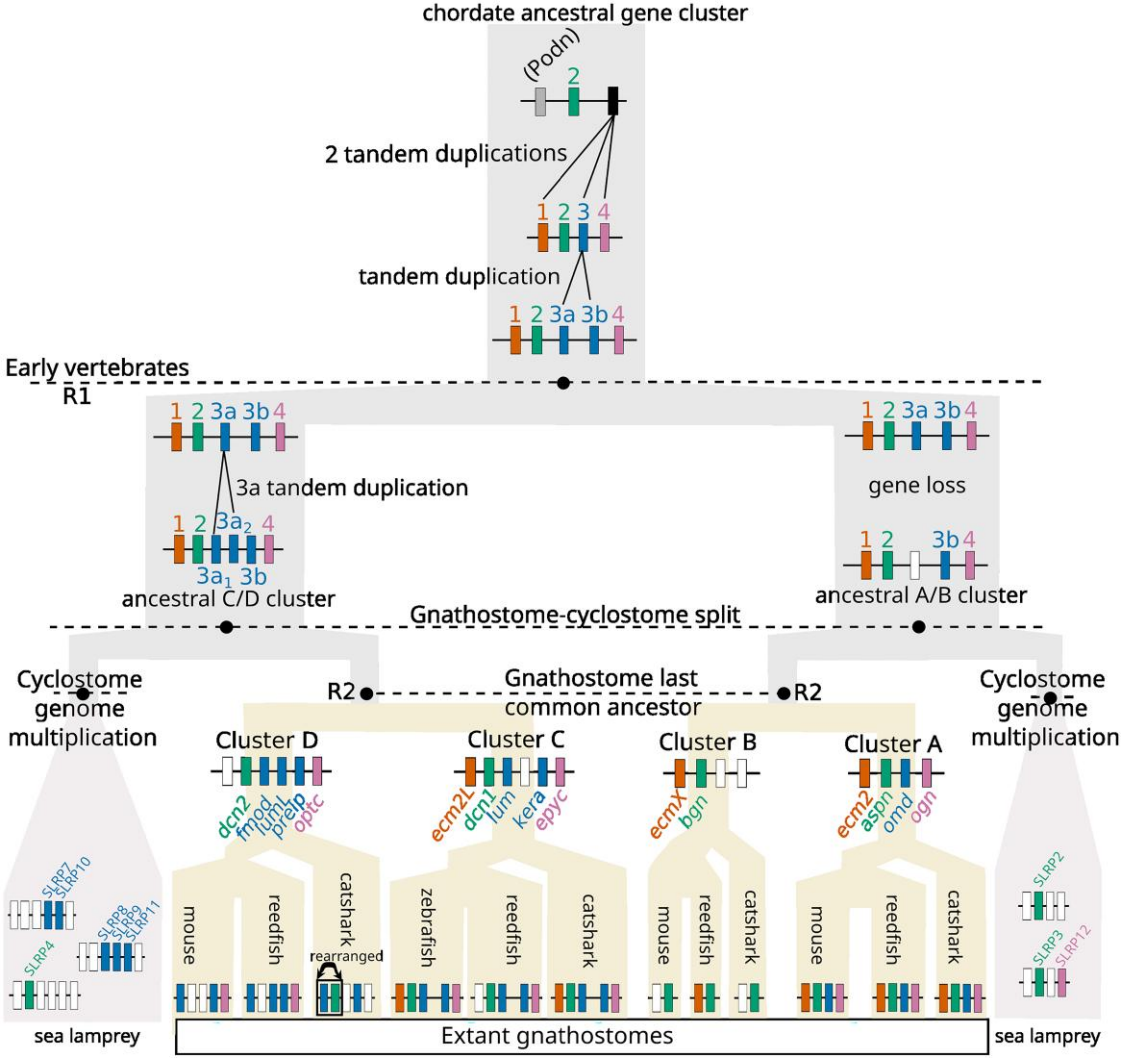
Hypothesized scenario for the amplification of the canonical SLRP gene family in chordates with regard to the R1 (genome duplication in a common ancestor to cyclostomes and gnathostomes) and successive genome multiplications in either the gnathostome (R2) or the cyclostome lineage. Only selected sea lamprey SLRP loci are shown (see Main text for details). Color code similar to Fig. 1. White rectangle indicates hypothesized gene loss. Loci not to scale.

Chondrichthyans and cyclostomes have proven highly valuable for inferring general and specific properties regarding the evolution of vertebrate gene families (Suzuki *et al*. 2017; Smith *et al*. 2018; Debiais-Thibaud *et al*. 2019; Leurs *et al*. 2022). Using chondrichthyan genome data, we show the clustering of gnathostome canonical SLRPs along 4 different loci and the branching of paralogs in Clade 1–4 phylogenies are all congruent with the expected pattern for cluster multiplications by the 2R whole-genome duplications (Dehal and Boore 2005) following a [(cluster A, cluster B), (cluster C, cluster D)] relation (Fig. 10). For instance, within Clade 2, *aspn* (cluster A) and *bgn* (cluster B) are more recent paralogs, while *dcn1* (cluster C) and *dcn2* (cluster D) are more recent paralogs (Fig. 3). This pattern is also visible for Clades 1 and 4 if we consider gene loss for the sister genes to *ecm2L* and *ogn,* respectively (Figs. 2 and 6). However, the observed topology for Clade 3 cannot be explained using only 2 rounds of whole-genome duplication and gene loss. To explain the several additional paralogs of Clade 3 observed in clusters C and D, we propose a most parsimonious scenario where 2 events of tandem duplication occurred, the first one before the first round of whole-genome duplication (R1 event) and the other one before the split between gnathostomes and cyclostomes (Fig. 10). Before the gnathostome second round of whole-genome duplication, one ancestral cluster with 4 SLRPs duplicated into clusters A and B, and another ancestral cluster with 6 SLRP paralogs gave rise to clusters C and D (Fig. 10). Subsequent gene loss (and the 3R additional whole-genome duplication in teleosts) would explain the genomic organization of extant gnathostomes and the inferred phylogeny. A recent study showed a clear case of clustered, tandemly duplicated genes diversifying through the 2R-event in the zone neighboring SLRPs with similar [(A,B), (C,D)] relationship (Ocampo Daza *et al*. 2022), making the excellent conservation of the gnathostome SLRP loci a wider characteristic of a whole chromosomal section.

In addition to gnathostome data, we identified 12 lamprey and 7 hagfish sequences as canonical SLRPs, both from their grouping with gnathostome canonical SLRPs into Clades 1–4, and supported by the presence of an LRRCE motif. Their exact phylogenetic relationship to each of the gnathostome paralogs is mostly strongly supported (Figs. 2–6). Lamprey sequences were found as smaller gene tandems, composed of mixes of genes belonging to Clades 1 and 3, or Clades 2 and 4, or several members of Clade 3 with a member of Clade 2 (Fig. 7). These characteristics are similar to gnathostome clusters, and support a scenario where tandem duplications that gave rise to the 4 SLRP clades predated the cyclostome/gnathostome divergence (Fig. 10). The SLRP clusters identified in extant species would then all derive from a single five-gene vertebrate ancestral cluster that underwent the 1R event, and subsequently duplicated again through the parallel genome duplication histories of gnathostomes and cyclostomes (Fig. 10). The cyclostome group (*SLRP2* and *SLRP3*) is sister to the gnathostome (*Bgn* and *Aspn*), supporting their shared evolution from the ancestral A/B cluster. Similarly, a robust phylogenetic relationship between the cyclostome SLRP5 and the gnathostome Ecm2 (Fig. 2) suggests a descendance from the ancestral A/B cluster. However, *SLRP5* is found in shared synteny with *SLRP6* (Fig. 7) which is phylogenetically related to the Lum and Fmod gnathostomes genes (Fig. 4) which are descendants of the ancestral C/D cluster (Fig. 4). This observation comes in contradiction to the proposed evolutionary scenario and may be the result of chromosomal rearrangements in cyclostomes where SLRP clusters appear much less well conserved than in gnathostomes, as also exemplified with the hagfish genomic data (Fig. 7). The evolutionary scenario accounting for Clade 3 topology involves tandem duplications in addition to the gnathostome 2 rounds of duplications: one of these tandem duplications produces: (i) the ancestor for the Omd and the (Kera, Prelp) group (Clade 3b, Fig. 5, duplicate 3b in Fig. 10); (ii) the other copy (duplicate 3a in Fig. 10) will undergo an additional tandem duplication to generate a series of 3 Clade 3 genes on the ancestral C/D cluster. The complete series of hypothetical gene duplication and losses are illustrated in Fig. 10.

### Evolution of SLRP transcriptional profile in gnathostome skeletal tissues

Two main types of SLRP expression patterns can be observed in the small-spotted catshark embryonic endoskeletal tissues: perichondrium- or chondrocyte-associated expressions. For instance, *bgn*, *dcn1,* and *lum* all show a perichondrium-associated expression pattern (Fig. 9, Table 2) congruent with these genes being described to interact with collagen I in mice (Chen and Birk 2013). The chondrocyte expression of *chad1*, *chad2*, *epyc*, *omd*, *ogn,* and *prelp* (Fig. 9, Table 2) is a conserved feature between the small-spotted catshark and bony fishes (Shinomura and Kimata 1992; Funderburgh *et al*. 1997; Wilda *et al*. 2000; Grover and Roughley 2001; Tillgren *et al*. 2015). Most bony fish SLRPs are also components of the bone matrix, associated with type I collagen fibers, and are expressed by osteoblasts [Bgn, Dcn1: (Kamiya *et al*. 2001); Aspn, Lum, Epyc, Ogn: (Khayal *et al*. 2018); Omd, (Sommarin *et al*. 1998); Prelp: (Li *et al*. 2016); Chad1: (Shen *et al*. 1998)] despite Chad2 (previously named Chondroadherin Like) was detected expressed in cartilage but not bone cells (Tillgren *et al*. 2015). These observations suggest a large part of SLRPs (canonical and non-canonical) were involved in specific skeletal and connective tissues already in the last common ancestor of extant gnathostomes, in particular in cartilage. The single report of SLRP gene expression in cyclostomes was in the hagfish *Eptatretus burgeri* (Ota *et al*. 2013) where one Class I SLRP gene (MgSLRP4 is the most similar to this sequence) was found expressed in mesenchymal and cartilaginous cells. Comparable expression data in lamprey species, although challenging, would shed light on the potentially vertebrate-wide shared function of SLRPs in either cartilaginous or mesenchymal/perichondrial tissues, resulting from the evolution of skeletal tissues in early vertebrates.

**Table 2.** Compared expression patterns of SLRP genes in mineralized tissues.

		chondrocytes		perichondrium		osteoblasts		odontoblasts		ameloblasts	
		chondr	osteich	chondr	osteich	chondr	osteich	chondr	osteich	chondr	osteich
Clade 2	*aspn*	+	−	−	+	*na*	+	−	+	−	
	*bgn*	−	+	+	+	*na*	+	−	+	+	+
	*dcn1*	−	+	+	+	*na*	+	+	+	−	−
Clade 3	*lum*	−	+	+	+	*na*	+	+	+	+	
	*omd*	+	+	−		*na*	+	−	+	+	+
	*prelp*	+	+	−		*na*	+	−		−	
Clade 4	*epyc*	+	+	−		*na*	+	−		−	
	*ogn*	+	+	−		*na*	+	−	+	+	
	*chad1*	+	+	−		*na*	+	−		−	
	*chad2*	+	+	−		*na*	−	−		−	

osteich: osteichhyan species (see main text for references) and chondr: chondrichthyan species as exemplified by the small-spotted catshark (this study); na: non-applicable; empty boxes for missing data.

Within gnathostomes, a major exception to this conservation status is *aspn*, which was shown to be expressed in the perichondrium but not in differentiated cartilage (Henry *et al*. 2001) and to interact with Collagen type I in mice (Kalamajski *et al*. 2009) (Table 2). In the small-spotted catshark however, *aspn* was only detected in chondrocytes and not in the perichondrium, suggesting divergent evolution of *aspn* expression patterns. In addition, the small-spotted catshark Omd protein sequence is one of the most divergent SLRPs when comparing it to its bony fish ortholog: it showed no glycosaminoglycan attachment sites but 6 N-glycosylation sites; there were no detected putative tyrosine sulfation sites while several are found in bony fishes; no acidic C-terminal region was found. Its expression was located in chondrocytes in the embryonic small-spotted catshark while it has been detected in mice osteoblasts (Ninomiya *et al*. 2007) and in rat fetal femur bone with a function in binding hydroxyapatite (Wendel *et al*. 1998). Since chondrichthyans lost the ability to make bone and have novel modes of mineralization (Seidel *et al*. 2016), evolution of the *aspn* and *omd* expression patterns and sequences might be linked to this phenotypic evolution. Conversely, the *bgn* and *lum* genes are expressed in mice chondrocytes and known to interact with Collagen II and Aggrecan (Wilda *et al*. 2000; Chen and Birk 2013) but they were not detectable in the cartilage in the small-spotted catshark (Fig. 9). Functional studies on these 4 genes may shed light on protein evolution events that correlate with the lineage-specific evolution of skeleton in extant gnathostomes.

Expression in small-spotted catshark embryonic exoskeletal tissues in this study was also focused on dermal denticles that develop similarly to teeth (Debiais-Thibaud *et al*. 2015). The observed patterns in the small-spotted catshark can be ameloblast-associated (*bgn*, o*gn*, and *omd*), odontoblast-associated (*dcn1*) or both (*lum*) (Fig. 9, Table 2). In mice teeth, *bgn* and *omd* were detected in both ameloblasts and odontoblasts while *aspn*, *dcn1*, *lum*, *ogn,* and *fmod* were only found in odontoblasts (Buchaille *et al*. 2000; Matsuura *et al*. 2001; Hou *et al*. 2012; Houari *et al*. 2014; Randilini *et al*. 2020). Therefore, we can speculate on a conserved function of *bgn* and *omd* in enamel/enameloid formation and of *dcn1* and *lum* in dentin formation. Odontoblasts, the dentine-producing cells, show more differences in SLRP expression between bony and cartilaginous fishes than ameloblasts, which is surprising as dentine is considered a stable tissue in vertebrates, while the evolution of extant forms of enamel and enameloid are considered more derived tissues (Kawasaki and Weiss 2008; Leurs *et al*. 2022). The odontoblasts are thought to be very similar to osteocytes in their differentiation and secretion pathways and again, the lack of *aspn* expression in small-spotted catshark odontoblasts might be another outcome of bone loss. To obtain a general trend in the evolution of exoskeleton cell types and tissues, a much denser characterization of genes expressed in teeth or scales in a variety of bony and cartilaginous fishes is necessary.

In contrast to other SLRPs, *ecm2L* and *kera* expression is enriched in very early embryonic stages (Table 1). Protein motifs and expression of *kera* are quite similar to its ortholog in bony fishes, which has been shown to be expressed very early during chick embryogenesis with possible roles in neural-crest cell migration (Conrad and Conrad 2003). We show that *ecm2L* was lost in amniotes and that the small-spotted catshark ortholog is very peculiar since we found 18 putative N-glycosylation sites before the LRRs. Together, these results suggest that Kera and Ecm2L may be important targets for future studies of SLRP involvement in the earliest step of skeletal development, through their potential role in neural-crest cell migration.

### Specific SLRP evolutionary trends in cartilaginous species

In our species sampling, *dcn2* was present only in chondrichthyans and inferred to be lost in all other gnathostome lineages. Screening of the protein motifs showed high similarity with its paralog Dcn1 which is itself very similar to its bony fish orthologs (Kalamajski and Oldberg 2010; Low *et al*. 2021), suggesting functional redundancy between these genes. However, *dcn2* expression enrichment in the small-spotted catshark kidney is not found for *dcn1*, which may be a functional reason for its maintenance in chondrichthyan genomes.

An enrichment of expression for *ecm2*, *bgn*, and *prelp* was detected in the ampullae of Lorenzini, which are sensory organs that can detect electromagnetic fields and temperature gradients and are filled with a keratan sulfate-rich gel. Previous studies on ampullae of Lorenzini did not identify the proteoglycans to which these keratan sulfate chains might be linked to (Zhang *et al*. 2018; Melrose 2019), but specifically *lum*, *ogn*, and *prelp* have been reported to carry keratan sulfate in mammals (Funderburgh *et al*. 1997; Hultgårdh-Nilsson *et al*. 2015; Iozzo and Schaefer 2015) and are highly expressed in the ampullae of Lorenzini (TPM > 50, Table 1). Predicted N-glycosylation and glycosaminoglycan attachment sites in the small-spotted catshark SLRP sequences were on conserved positions, including those where keratan sulfate attachment was shown in mammals (Low *et al*. 2021) (Supplementary Table 3). Therefore, *lum*, *ogn,* and *prelp* are excellent candidates for structural SLRPs involved in the secretion of the specialized gel of the ampullae of Lorenzini in chondrichthyan fishes. More surprising are *bgn* and *ecm2* expressions in the ampullae of Lorenzini. The small-spotted catshark Ecm2 is divergent when compared to its bony fish ortholog: it is predicted to have 5 possibly sulfated tyrosines in the N-terminal region (none predicted in mice), 4 putative glycosaminoglycan attachment sites (only one in mice) and 3 N-glycosylation sites (none in mice), all possible sites for keratan sulfate attachment. This SLRP might carry keratan sulfate chains in cartilaginous fishes, but these results are highly speculative since Ecm proteins have been very poorly studied in bony fishes, and our data are only predictions which are more permissive than the actual observation of glycosaminoglycan attachment (Kalamajski and Oldberg 2010)⁠⁠.

## Conclusion

Here, we characterized the repertoire of SLRPs in vertebrates by including cartilaginous fish, cyclostome and cephalochordate sequence data. The relative genomic stability of clustered SLRP genes in gnathostomes might be related to the observation of conserved transcriptional profiles suggesting high developmental constraints on the SLRP proteins, notably in relation to skeletal development and homeostasis. Our data support that several key features that evolved in early vertebrates (endo- and exoskeleton, sensory organs) depend on the expression of several members of the SLRP gene family. A thorough characterization of glycosaminoglycan chain linkage of chondrichthyan and other vertebrate SLRPs is now critical to better understand lineage-derived and ancestral features associated with the function of these proteoglycans.

## Supplementary Material

jkaf003_Supplementary_Data

## Data Availability

The authors affirm that all data necessary for confirming the conclusions of the article are present within the article, figures, and tables or through public databases as cited in Supplementary Table 1. Supplemental material available at G3 online.

## References

[jkaf003-B1] Altschul SF , Gish W, Miller W, Myers EW, Lipman DJ. 1990. Basic local alignment search tool. J Mol Biol. 215(3):403–410. 10.1016/S0022-2836(05)80360-2. 2231712

[jkaf003-B2] Bej A , Sahoo BR, Swain B, Basu M, Jayasankar P, Samanta M. 2014. LRRsearch: an asynchronous server-based application for the prediction of leucine-rich repeat motifs and an integrative database of NOD-like receptors. Comput Biol Med. 53:164–170. 10.1016/j.compbiomed.2014.07.016. 25150822

[jkaf003-B3] Berio F , Broyon M, Enault S, Pirot N, López-Romero FA, Debiais-Thibaud M. 2021. Diversity and evolution of mineralized skeletal tissues in chondrichthyans. Front Ecol Evol. 9:660767. 10.3389/fevo.2021.660767.

[jkaf003-B4] Boskey AL . 2010. The biochemistry of bone. In: Osteoporosis in Men. Elsevier. p. 3–13. 10.1016/B978-0-12-374602-3.00001-8.

[jkaf003-B5] Buchaille R , Couble ML, Magloire H, Bleicher F. 2000. Expression of the small leucine-rich proteoglycan osteoadherin/osteomodulin in human dental pulp and developing rat teeth. Bone. 27(2):265–270. 10.1016/S8756-3282(00)00310-0. 10913920

[jkaf003-B6] Chen S , Birk DE. 2013. The regulatory roles of small leucine-rich proteoglycans in extracellular matrix assembly. FEBS J. 280(10):2120–2137. 10.1111/febs.12136. 23331954 PMC3651807

[jkaf003-B7] Conrad AH , Conrad GW. 2003. The keratocan gene is expressed in both ocular and non-ocular tissues during early chick development. Matrix Biol. 22(4):323–337. 10.1016/S0945-053X(03)00039-8. 12935817

[jkaf003-B8] Costa RA , Martins RST, Capilla E, Anjos L, Power DM. 2018. Vertebrate SLRP family evolution and the subfunctionalization of osteoglycin gene duplicates in teleost fish. BMC Evol Biol. 18(1):191. 10.1186/s12862-018-1310-2. 30545285 PMC6293640

[jkaf003-B9] Debiais-Thibaud M , Chiori R, Enault S, Oulion S, Germon I, Martinand-Mari C, Casane D, Borday-Birraux V. 2015. Tooth and scale morphogenesis in shark: an alternative process to the mammalian enamel knot system. BMC Evol Biol. 15:292. 10.1186/s12862-015-0557-0. 26704180 PMC4690397

[jkaf003-B10] Debiais-Thibaud M , Simion P, Ventéo S, Muñoz D, Marcellini S, Mazan S, Haitina T. 2019. Skeletal mineralization in association with type X collagen expression is an ancestral feature for jawed vertebrates. Mol Biol Evol. 36(10):2265–2276. 10.1093/molbev/msz145. 31270539 PMC6759074

[jkaf003-B11] de Castro E , Sigrist CJA, Gattiker A, Bulliard V, Langendijk-Genevaux PS, Gasteiger E, Bairoch A, Hulo N. 2006. ScanProsite: detection of PROSITE signature matches and ProRule-associated functional and structural residues in proteins. Nucleic Acids Res. 34(Web Server):W362–W365. 10.1093/nar/gkl124. 16845026 PMC1538847

[jkaf003-B12] Dehal P , Boore JL. 2005. Two rounds of whole genome duplication in the ancestral vertebrate. PLoS Biol. 3(10):e314. 10.1371/journal.pbio.0030314. 16128622 PMC1197285

[jkaf003-B13] Enault S , Adnet S, Debiais-Thibaud M. 2016. Skeletogenesis during the late embryonic development of the catshark *Scyliorhinus canicula* (Chondrichthyes; Neoselachii). M3. 1:e2. 10.18563/m3.1.4.e2.

[jkaf003-B14] Funderburgh JL , Corpuz LM, Roth MR, Funderburgh ML, Tasheva ES, Conrad GW. 1997. Mimecan, the 25-kDa corneal keratan sulfate proteoglycan, is a product of the gene producing osteoglycin. J Biol Chem. 272(44):28089–28095. 10.1074/jbc.272.44.28089. 9346963

[jkaf003-B15] Grover J , Roughley PJ. 2001. Characterization and expression of murine PRELP. Matrix Biol. 20(8):555–564. 10.1016/S0945-053X(01)00165-2. 11731272

[jkaf003-B16] Guindon S , Dufayard J-F, Lefort V, Anisimova M, Hordijk W, Gascuel O. 2010. New algorithms and methods to estimate maximum-likelihood phylogenies: assessing the performance of PhyML 3.0. Syst Biol. 59(3):307–321. 10.1093/sysbio/syq010. 20525638

[jkaf003-B17] Hardingham T . 2006. Proteoglycans and glycosaminoglycans. In:Dynamics of Bone and Cartilage Metabolism. Elsevier. p. 85–98. 10.1016/B978-012088562-6/50006-6.

[jkaf003-B18] Henry SP , Takanosu M, Boyd TC, Mayne PM, Eberspaecher H, Zhou W, de Crombrugghe B, Höök M, Mayne R. 2001. Expression pattern and gene characterization of asporin. a newly discovered member of the leucine-rich repeat protein family. J Biol Chem. 276(15):12212–12221. 10.1074/jbc.M011290200. 11152695

[jkaf003-B19] Hoang DT , Chernomor O, von Haeseler A, Minh BQ, Vinh LS. 2018. UFBoot2: improving the ultrafast bootstrap approximation. Mol Biol Evol. 35(2):518–522. 10.1093/molbev/msx281. 29077904 PMC5850222

[jkaf003-B20] Homma S , Shimada T, Hikake T, Yaginuma H. 2009. Expression pattern of LRR and Ig domain-containing protein (LRRIG protein) in the early mouse embryo. Gene Expr Patterns. 9(1):1–26. 10.1016/j.gep.2008.09.004. 18848646

[jkaf003-B21] Hou C , Liu ZX, Tang KL, Wang MG, Sun J, Wang J, Li S. 2012. Developmental changes and regional localization of Dspp, Mepe, Mimecan and Versican in postnatal developing mouse teeth. J Mol Hist. 43(1):9–16. 10.1007/s10735-011-9368-9. 22042093

[jkaf003-B22] Houari S , Wurtz T, Ferbus D, Chateau D, Dessombz A, Berdal A, Babajko S. 2014. Asporin and the mineralization process in fluoride-treated rats. J Bone Miner Res. 29(6):1446–1455. 10.1002/jbmr.2153. 24967458

[jkaf003-B23] Hultgårdh-Nilsson A , Borén J, Chakravarti S. 2015. The small leucine-rich repeat proteoglycans in tissue repair and atherosclerosis. J Intern Med. 278(5):447–461. 10.1111/joim.12400. 26477596 PMC4616156

[jkaf003-B24] Iozzo RV , Schaefer L. 2015. Proteoglycan form and function: a comprehensive nomenclature of proteoglycans. Matrix Biol. 42:11–55. 10.1016/j.matbio.2015.02.003. 25701227 PMC4859157

[jkaf003-B25] Jaillon O , Aury J-M, Brunet F, Petit J-L, Stange-Thomann N, Mauceli E, Bouneau L, Fischer C, Ozouf-Costaz C, Bernot A, et al 2004. Genome duplication in the teleost fish *Tetraodon nigroviridis* reveals the early vertebrate proto-karyotype. Nature. 431(7011):946–957. 10.1038/nature03025. 15496914

[jkaf003-B26] Kalamajski S , Aspberg A, Lindblom K, Heinegård D, Oldberg Å. 2009. Asporin competes with decorin for collagen binding, binds calcium and promotes osteoblast collagen mineralization. Biochem J. 423(1):53–59. 10.1042/BJ20090542. 19589127

[jkaf003-B27] Kalamajski S , Oldberg Å. 2010. The role of small leucine-rich proteoglycans in collagen fibrillogenesis. Matrix Biol. 29(4):248–253. 10.1016/j.matbio.2010.01.001. 20080181

[jkaf003-B28] Kalyaanamoorthy S , Minh BQ, Wong TKF, von Haeseler A, Jermiin LS. 2017. ModelFinder: fast model selection for accurate phylogenetic estimates. Nat Methods. 14(6):587–589. 10.1038/nmeth.4285. 28481363 PMC5453245

[jkaf003-B29] Kamiya N , Shigemasa K, Takagi M. 2001. Gene expression and immunohistochemical localization of decorin and biglycan in association with early bone formation in the developing mandible. J Oral Sci. 43(3):179–188. 10.2334/josnusd.43.179. 11732738

[jkaf003-B30] Kasahara M , Naruse K, Sasaki S, Nakatani Y, Qu W, Ahsan B, Yamada T, Nagayasu Y, Doi K, Kasai Y, et al 2007. The medaka draft genome and insights into vertebrate genome evolution. Nature. 447(7145):714–719. 10.1038/nature05846. 17554307

[jkaf003-B31] Katoh K , Standley DM. 2016. A simple method to control over-alignment in the MAFFT multiple sequence alignment program. Bioinformatics. 32(13):1933–1942. 10.1093/bioinformatics/btw108. 27153688 PMC4920119

[jkaf003-B32] Kawasaki K , Weiss KM. 2008. SCPP Gene evolution and the dental mineralization Continuum. J Dent Res. 87(6):520–531. 10.1177/154405910808700608. 18502959

[jkaf003-B33] Khayal LA , Grünhagen J, Provazník I, Mundlos S, Kornak U, Robinson PN, Ott C-E. 2018. Transcriptional profiling of murine osteoblast differentiation based on RNA-seq expression analyses. Bone. 113:29–40. 10.1016/j.bone.2018.04.006. 29653293

[jkaf003-B34] Kumar M , Gouw M, Michael S, Sámano-Sánchez H, Pancsa R, Glavina J, Diakogianni A, Valverde JA, Bukirova D, Čalyševa J, et al 2019. ELM—the eukaryotic linear motif resource in 2020. Nucleic Acids Res. 48(D1):D296–D306. 10.1093/nar/gkz1030. PMC7145657 31680160

[jkaf003-B35] Leurs N , Martinand-Mari C, Marcellini S, Debiais-Thibaud M. 2022. Parallel evolution of ameloblastic *scpp* genes in bony and cartilaginous vertebrates. Mol Biol Evol. 39(5):msac099. 10.1093/molbev/msac099. 35535508 PMC9122587

[jkaf003-B36] Leurs N , Martinand-Mari C, Ventéo S, Haitina T, Debiais-Thibaud M. 2021. Evolution of matrix Gla and bone Gla protein genes in jawed vertebrates. Front Genet. 12:620659. 10.3389/fgene.2021.620659. 33790944 PMC8006282

[jkaf003-B37] Li H , Cui Y, Luan J, Zhang X, Li C, Zhou X, Shi L, Wang H, Han J. 2016. PRELP (proline/arginine-rich end leucine-rich repeat protein) promotes osteoblastic differentiation of preosteoblastic MC3T3-E1 cells by regulating the β-catenin pathway. Biochem Biophys Res Commun. 470(3):558–562. 10.1016/j.bbrc.2016.01.106. 26809092

[jkaf003-B38] Low SWY , Connor TB, Kassem IS, Costakos DM, Chaurasia SS. 2021. Small leucine-rich proteoglycans (SLRPs) in the retina. Int J Mol Sci. 22(14):7293. 10.3390/ijms22147293. 34298915 PMC8305803

[jkaf003-B39] Marlétaz F , Timoshevskaya N, Timoshevskiy VA, Parey E, Simakov O, Gavriouchkina D, Suzuki M, Kubokawa K, Brenner S, Smith JJ, et al 2024. The hagfish genome and the evolution of vertebrates. Nature. 627(8005):811–820. 10.1038/s41586-024-07070-3. 38262590 PMC10972751

[jkaf003-B40] Matsushima N , Miyashita H, Kretsinger RH. 2021. Sequence features, structure, ligand interaction, and diseases in small leucine rich repeat proteoglycans. J Cell Commun Signal. 15(4):519–531. 10.1007/s12079-021-00616-4. 33860400 PMC8642563

[jkaf003-B41] Matsuura T , Duarte WR, Cheng H, Uzawa K, Yamauchi M. 2001. Differential expression of decorin and biglycan genes during mouse tooth development. Matrix Biol. 20(5–6):367–373. 10.1016/S0945-053X(01)00142-1. 11566271

[jkaf003-B42] Mayeur H , Leyhr J, Mulley J, Leurs N, Michel L, Sharma K, Lagadec R, Aury J-M, Osborne OG, Mulhair P, et al 2024. The sensory shark: high-quality morphological, genomic and transcriptomic data for the small-spotted catshark *Scyliorhinus canicula* reveal the molecular bases of sensory organ evolution in jawed vertebrates. Mol Biol Evol. 41(12):msae246. 10.1093/molbev/msae246. PMC11979771 39657112

[jkaf003-B43] Melrose J . 2019. Functional consequences of keratan sulfate sulfation in electrosensory tissues and in neuronal regulation. Adv Biosyst. 3(4):1800327. 10.1002/adbi.201800327. 32627425

[jkaf003-B44] Mochida Y , Parisuthiman D, Kaku M, Hanai J, Sukhatme VP, Yamauchi M. 2006. Nephrocan, a novel member of the small leucine-rich repeat protein family, is an inhibitor of transforming growth factor-β signaling. J Biol Chem. 281(47):36044–36051. 10.1074/jbc.M604787200. 16990280

[jkaf003-B45] Monigatti F , Gasteiger E, Bairoch A, Jung E. 2002. The sulfinator: predicting tyrosine sulfation sites in protein sequences. Bioinformatics. 18(5):769–770. 10.1093/bioinformatics/18.5.769. 12050077

[jkaf003-B46] Nakatani Y , Shingate P, Ravi V, Pillai NE, Prasad A, McLysaght A, Venkatesh B. 2021. Reconstruction of proto-vertebrate, proto-cyclostome and proto-gnathostome genomes provides new insights into early vertebrate evolution. Nat Commun. 12(1):4489. 10.1038/s41467-021-24573-z. 34301952 PMC8302630

[jkaf003-B47] Nguyen L-T , Schmidt HA, von Haeseler A, Minh BQ. 2015. IQ-TREE: a fast and effective stochastic algorithm for estimating maximum-likelihood phylogenies. Mol Biol Evol. 32(1):268–274. 10.1093/molbev/msu300. 25371430 PMC4271533

[jkaf003-B48] Nikitovic D , Aggelidakis J, Young MF, Iozzo RV, Karamanos NK, Tzanakakis GN. 2012. The biology of small leucine-rich proteoglycans in bone pathophysiology. J Biol Chem. 287(41):33926–33933. 10.1074/jbc.R112.379602. 22879588 PMC3464503

[jkaf003-B49] Ninomiya K , Miyamoto T, Imai J, Fujita N, Suzuki T, Iwasaki R, Yagi M, Watanabe S, Toyama Y, Suda T. 2007. Osteoclastic activity induces osteomodulin expression in osteoblasts. Biochem Biophys Res Commun. 362(2):460–466. 10.1016/j.bbrc.2007.07.193. 17714690

[jkaf003-B50] Ocampo Daza D , Bergqvist CA, Larhammar D. 2022. The evolution of oxytocin and vasotocin receptor genes in jawed vertebrates: a clear case for gene duplications through ancestral whole-genome duplications. Front Endocrinol. 12:792644. 10.3389/fendo.2021.792644. PMC8851675 35185783

[jkaf003-B51] Ohno S . 1970. The enormous diversity in genome sizes of fish as a reflection of Nature's extensive experiments with gene duplication. Trans Am Fish Soc. 99(1):120–130. 10.1577/1548-8659(1970)99<120:TEDIGS>2.0.CO;2.

[jkaf003-B52] Ota KG , Fujimoto S, Oisi Y, Kuratani S. 2013. Late development of hagfish vertebral elements. J Exp Zool B Mol Dev Evol. 320(3):129–139. 10.1002/jez.b.22489. 23401412 PMC3646255

[jkaf003-B53] Park H , Huxley-Jones J, Boot-Handford RP, Bishop PN, Attwood TK, Bella J. 2008. LRRCE: a leucine-rich repeat cysteine capping motif unique to the chordate lineage. BMC Genomics. 9:599. 10.1186/1471-2164-9-599. 19077264 PMC2637281

[jkaf003-B54] Randilini A , Fujikawa K, Shibata S. 2020. Expression, localization and synthesis of small leucine-rich proteoglycans in developing mouse molar tooth germ. Eur J Histochem. 64(1):3092. 10.4081/ejh.2020.3092. 32046476 PMC7029624

[jkaf003-B55] Root ZD , Allen C, Gould C, Brewer M, Jandzik D, Medeiros DM. 2022. A comprehensive analysis of fibrillar collagens in lamprey suggests a conserved role in vertebrate musculoskeletal evolution. Front Cell Dev Biol. 10:809979. 10.3389/fcell.2022.809979. 35242758 PMC8887668

[jkaf003-B56] Root ZD , Jandzik D, Allen C, Brewer M, Romášek M, Square T, Medeiros DM. 2021. Lamprey lecticans link new vertebrate genes to the origin and elaboration of vertebrate tissues. Dev Biol. 476:282–293. 10.1016/j.ydbio.2021.03.020. 33887266

[jkaf003-B57] Schaefer L , Iozzo RV. 2008. Biological functions of the small leucine-rich proteoglycans: from genetics to signal transduction. J Biol Chem. 283(31):21305–21309. 10.1074/jbc.R800020200. 18463092 PMC2490788

[jkaf003-B58] Seidel R , Lyons K, Blumer M, Zaslansky P, Fratzl P, Weaver JC, Dean MN. 2016. Ultrastructural and developmental features of the tessellated endoskeleton of elasmobranchs (sharks and rays). J Anat. 229(5):681–702. 10.1111/joa.12508. 27557870 PMC5055090

[jkaf003-B59] Shen Z , Gantcheva S, Månsson B, Heinegård D, Sommarin Y. 1998. Chondroadherin expression changes in skeletal development. Biochem J. 330(1):549–557. 10.1042/bj3300549. 9461555 PMC1219172

[jkaf003-B60] Shinomura T , Kimata K. 1992. Proteoglycan-Lb, a small dermatan sulfate proteoglycan expressed in embryonic chick epiphyseal cartilage, is structurally related to osteoinductive factor. J Biol Chem. 267(2):1265–1270. 10.1016/S0021-9258(18)48424-4. 1730648

[jkaf003-B61] Smith JJ , Timoshevskaya N, Ye C, Holt C, Keinath MC, Parker HJ, Cook ME, Hess JE, Narum SR, Lamanna F, et al 2018. The sea lamprey germline genome provides insights into programmed genome rearrangement and vertebrate evolution. Nat Genet. 50(2):270–277. 10.1038/s41588-017-0036-1. 29358652 PMC5805609

[jkaf003-B62] Sommarin Y , Wendel M, Shen Z, Hellman U, Heinegård D. 1998. Osteoadherin, a cell-binding keratan sulfate proteoglycan in bone, belongs to the family of leucine-rich repeat proteins of the extracellular matrix. J Biol Chem. 273(27):16723–16729. 10.1074/jbc.273.27.16723. 9642227

[jkaf003-B63] Suzuki A , Komata H, Iwashita S, Seto S, Ikeya H, Tabata M, Kitano T. 2017. Evolution of the RH gene family in vertebrates revealed by brown hagfish (*Eptatretus atami*) genome sequences. Mol Phylogenet Evol. 107:1–9. 10.1016/j.ympev.2016.10.004. 27746317

[jkaf003-B64] Tillgren V , Ho JCS, Önnerfjord P, Kalamajski S. 2015. The novel small leucine-rich protein chondroadherin-like (CHADL) is expressed in cartilage and modulates chondrocyte differentiation. J Biol Chem. 290(2):918–925. 10.1074/jbc.M114.593541. 25451920 PMC4294519

[jkaf003-B65] Wendel M , Sommarin Y, Heinegård D. 1998. Bone matrix proteins: isolation and characterization of a novel cell-binding keratan sulfate proteoglycan (osteoadherin) from bovine bone. J Cell Biol. 141(3):839–847. 10.1083/jcb.141.3.839. 9566981 PMC2132750

[jkaf003-B66] Wilda M , Bächner D, Just W, Geerkens C, Kraus P, Vogel W, Hameister H. 2000. A comparison of the expression pattern of five genes of the family of small leucine-rich proteoglycans during mouse development. J Bone Miner Res. 15(11):2187–2196. 10.1359/jbmr.2000.15.11.2187. 11092399

[jkaf003-B67] Zappia J , Joiret M, Sanchez C, Lambert C, Geris L, Muller M, Henrotin Y. 2020. From translation to protein degradation as mechanisms for regulating biological functions: a review on the SLRP family in skeletal tissues. Biomolecules. 10(1):80. 10.3390/biom10010080. 31947880 PMC7023458

[jkaf003-B68] Zhang X , Xia K, Lin L, Zhang F, Yu Y, St. Ange K, Han X, Edsinger E, Sohn J, Linhardt RJ. 2018. Structural and functional components of the skate sensory organ ampullae of lorenzini. ACS Chem Biol. 13(6):1677–1685. 10.1021/acschembio.8b00335. 29708722

