# Supplementary material for: The vertebrate small leucine-rich proteoglycans: amplification of a clustered gene family and evolution of their transcriptional profile in jawed vertebrates
Source: G3 (Bethesda). 2025 Jan 8;15(3):jkaf003. doi: 10.1093/g3journal/jkaf003 (PMC11917481; doi:10.1093/g3journal/jkaf003)

Supplementary Figure S2: qPCR data

qPCR relative expression levels are normalized internally for each gene, so values cannot be compared between genes, but the global variation of expression over time can. The SLRP relative expression was in general down -regulated over the course of tissue differentiation except in the case of *omd*, *lum*, *fmod* and *podn*.

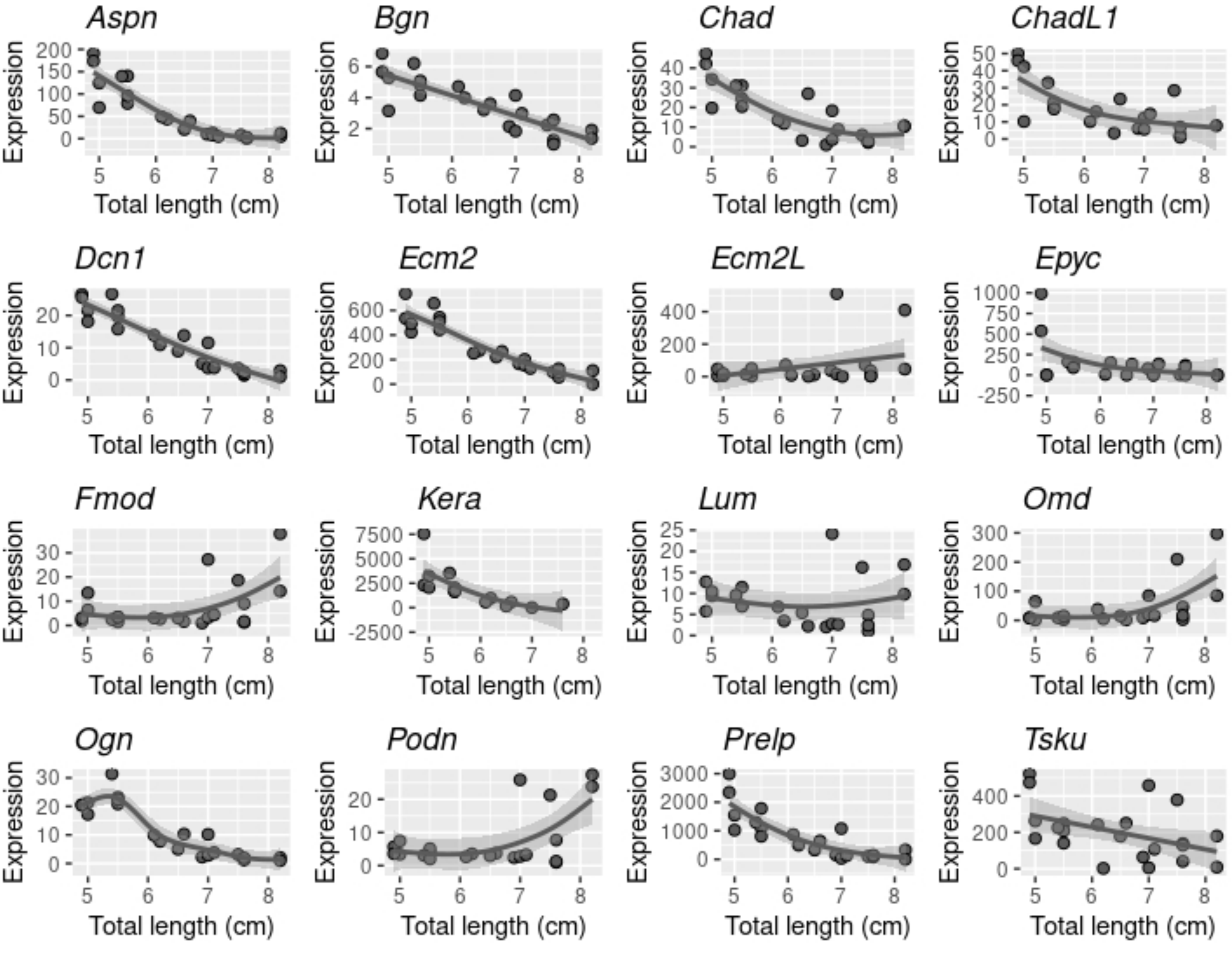

Supplement: jkaf003_Supplementary_Data [file jkaf003_supplementary_data.zip › Supplemental_Figure_S2_G3-2024-405575.pdf]
