# Supplementary material for: The vertebrate small leucine-rich proteoglycans: amplification of a clustered gene family and evolution of their transcriptional profile in jawed vertebrates
Source: G3 (Bethesda). 2025 Jan 8;15(3):jkaf003. doi: 10.1093/g3journal/jkaf003 (PMC11917481; doi:10.1093/g3journal/jkaf003)
Supplement: jkaf003_Supplementary_Data [file jkaf003_supplementary_data.zip › Supplemental_Material_Legends_G3-2024-405575.docx]

**Supplemental material**

**Supplemental** **Table S1**: Accession numbers of all sequences used in phylogenetic reconstruction

**Supplemental** **Table S2**: Primer sequences used for qPCR and *in situ* hybridizations

**Supplemental** **Table S3**: Predicted SLRP protein domains for all small-spotted catshark SLRPs

**Supplemental** **Table S4**: Table of TPM values for SLRP genes in the small-spotted catshark, as extracted from the transcriptomic data published in Mayeur et al. 2024.

**Supplemental** **Table S5**: Table of Zscore values for SLRP genes in the small-spotted catshark, extracted from the transcriptomic data published in Mayeur et al. 2024

**Supplemental** **File S1**: Alignment used for the phylogenetic reconstruction

**Supplemental** **File S2**: Tree file generated in this study

**Supplemental** **Figure S1**: Full annotated phylogeny

**Supplemental Figure S2**: qPCR data for selected SLRP genes
