# Supplementary material for: The vertebrate small leucine-rich proteoglycans: amplification of a clustered gene family and evolution of their transcriptional profile in jawed vertebrates
Source: G3 (Bethesda). 2025 Jan 8;15(3):jkaf003. doi: 10.1093/g3journal/jkaf003 (PMC11917481; doi:10.1093/g3journal/jkaf003)
Supplement: jkaf003_Supplementary_Data [file Supplemental_Figure_S1_G3-2024-405575.pdf › Supplemental_Figure_S1_G3-2024-405575.pdf]

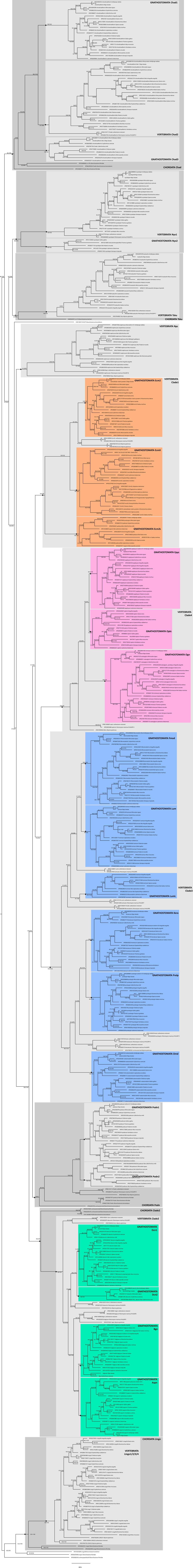
GNATHOSTOMATA Chad1
VERTEBRATA Chad2
GNATHOSTOMATA Chad3
CHORDATA Chad
VERTEBRATA Nyx1
VERTEBRATA Nyx2
VERTEBRATA Tsku
CHORDATA Tsku
VERTEBRATA Npn
GNATHOSTOMATA Ecm2
VERTEBRATA Clade1
GNATHOSTOMATA EcmX
GNATHOSTOMATA Ecm2L
GNATHOSTOMATA Epyc
VERTEBRATA Clade4
GNATHOSTOMATA Optc
GNATHOSTOMATA Ogn
GNATHOSTOMATA Fmod
GNATHOSTOMATA Lum
VERTEBRATA Clade3
GNATHOSTOMATA LumL
GNATHOSTOMATA Kera
GNATHOSTOMATA Prelp
GNATHOSTOMATA Omd
GNATHOSTOMATA Podn1
GNATHOSTOMATA Podn2
CHORDATA Podn
CHORDATA Clade2
VERTEBRATA Clade2
GNATHOSTOMATA Dcn1
GNATHOSTOMATA Dcn2
GNATHOSTOMATA Bgn
GNATHOSTOMATA Aspn
CHORDATA Lingo
VERTEBRATA Lingo1/2/3/4
0.1
